# New Quipazine Derivatives Active Against Drug-Resistant Oncogenic *Helicobacter pylori* Strains with Biofilm

**DOI:** 10.3390/ijms26135997

**Published:** 2025-06-22

**Authors:** Katarzyna Grychowska, Karolina Klesiewicz, Joanna Pęgiel, Agata Kuziak, Iwona Skiba-Kurek, Vittorio Canale, Gracjana Krzysiek-Mączka, Agata Ptak-Belowska, Kamil Piska, Paulina Koczurkiewicz-Adamczyk, Paweł Krzyżek, Tomasz Brzozowski, Paweł Zajdel, Elżbieta Karczewska

**Affiliations:** 1 Faculty of Pharmacy, Jagiellonian University Medical College, 9 Medyczna Str., 30-688 Kraków, Poland; joanna.1.pegiel@uj.edu.pl (J.P.); agata.kuziak@uj.edu.pl (A.K.); iwona.skiba@uj.edu.pl (I.S.-K.); vittorio.canale@uj.edu.pl (V.C.); kamil.piska@uj.edu.pl (K.P.); paulina.koczurkiewicz@uj.edu.pl (P.K.-A.); pawel.zajdel@uj.edu.pl (P.Z.); elzbieta.karczewska@uj.edu.pl (E.K.); 2 Faculty of Medicine, Jagiellonian University Medical College, 16 Grzegórzecka Str., 31-531 Kraków, Poland; gracjana.krzysiek-maczka@uj.edu.pl (G.K.-M.); agata.ptak-belowska@uj.edu.pl (A.P.-B.); tomasz.brzozowski@uj.edu.pl (T.B.); 3 Department of Microbiology, Faculty of Medicine, Wrocław Medical University, 4 Chałubińskiego Str., 50-368 Wroclaw, Poland; pawel.krzyzek@umw.edu.pl

**Keywords:** *Helicobacter pylori*, antibacterial activity, quipazine derivatives, biofilm, synergy, *H. pylori* CagA-positive strain

## Abstract

*Helicobacter pylori* (*H. pylori*) is regarded as a significant risk factor for gastritis, peptic ulcer disease, and gastric cancer. However, the increasing resistance of *H. pylori* strains has resulted in low eradication rates and ineffective treatments. Herein, we report on identification of a new quipazine derivative—compound **9c** (*N*-(3-chlorobenzyl)-2-(piperazin-1-yl)quinolin-4-amine), which displayed antibacterial properties (MIC range 2–4 µg/mL) against *H. pylori* CagA-positive reference strains associated with an increased risk of gastric cancer, including metronidazole-resistant ATCC 43504, clarithromycin-resistant ATCC 700684 and susceptible J99 strain, as well as clinical, multidrug-resistant isolate (3CML, resistant to clarithromycin, metronidazole and levofloxacin). Compound **9c** showed bacteriostatic activity (MBC/MIC ratio > 4), demonstrated antibiofilm-forming properties and prevented auto-aggregation of microbial cells. It also displayed an additive effect in ½ MIC (2 µg/mL) when administered with clarithromycin and/or metronidazole. Compound **9c** had no impact on gut microbiota reference strains of *S. aureus*, *E. coli*, *E. faecalis* and *L. paracasei* as well as no hemolytic activity against sheep erythrocytes. Finally, by reducing the viability of the SNU-1 human gastric cancer cell line (IC_50_ = 3.28 μg/mL), compound **9c** might offer important implications regarding the oncogenic characteristics of *cagA*+ *H. pylori* strains.

## 1. Introduction

*Helicobacter pylori* (*H. pylori*), a Gram-negative bacterium that colonizes the gastric epithelium, is considered one of the main etiological factors of gastritis and peptic ulcer disease [1,2]. Chronic infection with *H. pylori* strains carrying the *cag*A gene, that produces CagA oncoprotein, is the strongest risk factor for gastric cancer. Therefore, in 1994, the World Health Organization (WHO) classified *H. pylori* as a Class I carcinogen [3]. Eradication of *H. pylori* decreases the likelihood of gastric cancer mortality, underlining the crucial role of effective eradication therapy in mitigating the progression of carcinogenic processes [4,5]. Given the high prevalence of *H. pylori* infections worldwide (about 50%) and the production of the oncogenic CagA protein by approximately 60% of the strains that are the etiological factor of these infections, combating them becomes a serious clinical challenge [4,6,7].

The treatment of *H. pylori* involves combination therapy to increase drug efficacy and prevent the development of resistance mechanisms. The following treatment regimens are currently recommended for *H. pylori* infections: proton pump inhibitors (PPIs), bismuth salts and a combination of two or three chemotherapeutic agents selected from clarithromycin, metronidazole, levofloxacin, amoxicillin, tetracycline or rifabutin. [8,9]. However, resistance to antibiotics and chemotherapeutics, especially clarithromycin, that decreases the effectiveness of treatment by up to 80% constitutes a serious concern [10,11].

Therapeutic challenges may also arise from the ability of *H. pylori* strains to form biofilm. The multilayer biofilm structure enhances bacterial defense against antimicrobial agents and makes them highly resistant to drastic environmental changes including pH, temperature, oxygen deficiency and antibiotic exposure. Therefore, the elimination or dispersion of biofilm structures is considered a valuable strategy for *H. pylori* eradication [8].

**Figure 1:**
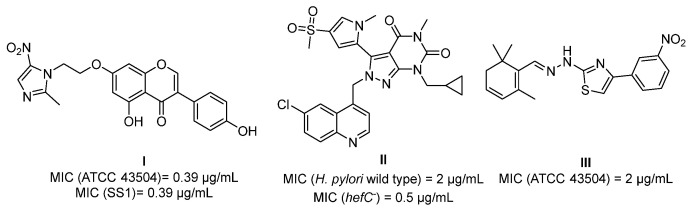
Selected chemotherapeutics active against *H. pylori*.

The development of agents targeted against *H. pylori* involves natural source-derived compounds (e.g., cinnamic acid, chroman, coumarin, shikimic acid derivatives) and synthetic derivatives originating from various chemical classes, e.g., nitro-group containing heterocycles (**I**), azabicyclic heterocycles (**II**) and hydrazone derivatives (**III**) (Figure 1) [12].

**Figure 2:**
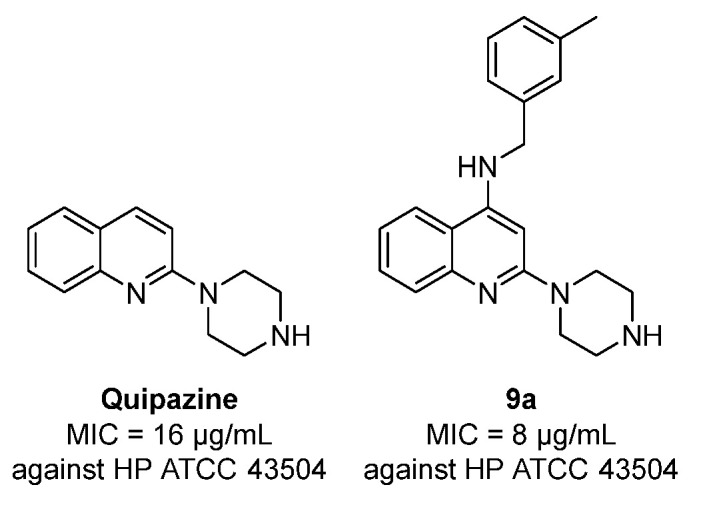
Quipazine does not exhibit measurable antibacterial activity against metronidazole-resistant *H. pylori* ATCC 43504 strain. However, the introduction of a 3-methylbenzylamine fragment improves activity (compound **9a**).

Drug repurposing, which involves finding new applications for already existing drugs, holds particular promise in addressing the problem of antimicrobial resistance. The screening of our in-house library of central nervous system-acting agents identified compound **9a**, a quipazine derivative, which displayed antibacterial properties against reference, metronidazole-resistant *H. pylori* strain ATCC 43504 (Figure 2).

This prompted us to design, synthesize and evaluate the in vitro antibacterial activity of new analogs of compound **9a**. The structural diversification involved the introduction of various substitution patterns in the benzylamine fragment to explore the influence of electronic (methyl group vs. chlorine atom) and regioisomeric effects (various positions of chlorine) on antibacterial activity. Additionally, the piperazine moiety was replaced with morpholine, and the quinoline scaffold was modified by the addition of methoxy groups. The latter modification, aimed at increasing antibacterial activity and safety profile, originated from the structure of fluoroquinolones (i.e., moxifloxacin, gatifloxacin) [13].

Antibacterial properties of obtained compounds were evaluated against four *H. pylori* strains, including reference strains resistant to metronidazole and clarithromycin (ATCC 43504 and ATCC 700684, respectively), and reference-susceptible strains harboring the *cag*A gene (J99, *cag*A+), associated with an increased risk of gastric cancer, and the clinical multi drug-resistant strain *cag*A+(3CML, resistant to clarithromycin, metronidazole and levofloxacin). The bactericidal/bacteriostatic activity of compound **9c** was determined, followed by the investigation of its ability to inhibit biofilm production. The selectivity of compound **9c** for *H. pylori* over selected bacteria strains, including components of gut microbiota, was analyzed. The synergistic activity of compound **9c** with clarithromycin and metronidazole showed its potential to enhance the efficacy of these drugs in overcoming drug resistance to *H. pylori* strains. Finally, the cytotoxic properties of compound **9c** in SNU-1 human gastric cancer cell line were assessed.

## 2. Results and Discussion

### 2.1. Chemistry

**Scheme 1:**
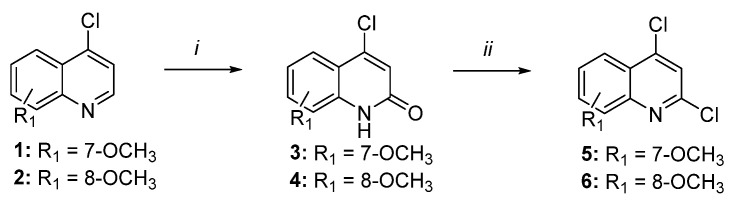
Reagents and conditions. (*i*) mCPBA, rt, 30 min, (yields 80–85%) (*ii*) POCl_3_, 150 °C, 1 h (65–70%).

The workflow began with the preparation of 7- and 8-methoxy-2,4-dichloroquinolines (**5**, **6**), which served as scaffolds for further structural modifications. For this purpose, 4-chloro-7-methoxyquinoline **1** and 4-chloro-8-methoxyquinoline **2** were oxidated using *meta*-chloroperoxybenzoic acid (mPCBA) to lactams **3** and **4**, followed by chlorination with phosphorus oxychloride to dichloroquinolines **5** and **6** (Scheme 1). The spectral data for compounds **3** and **5**, previously described in the literature [14], are in agreement with reported data.

**Scheme 2:**
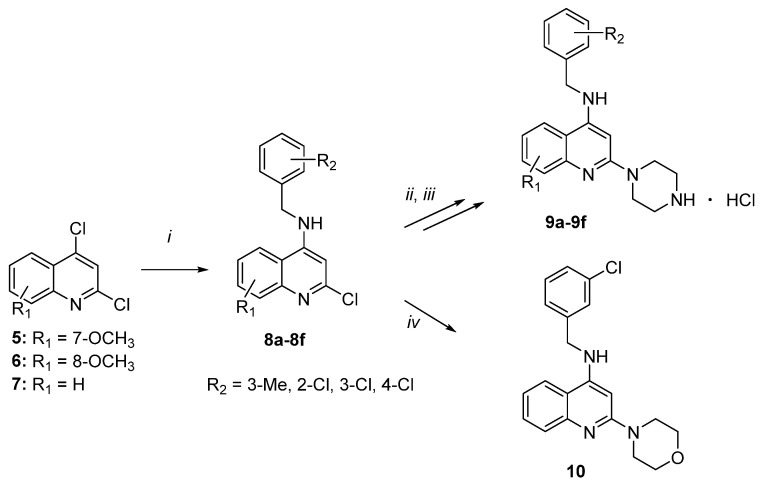
Reagents and conditions. (*i*) respective benzyl amine, DMSO, 140 °C, MW, 3 h, (yields 30–45%) (*ii*) Boc-piperazine, MeCN, 140 °C, MW, 7 h, (yields 45–65%) (*iii*) 6 M HCl/isopropanol, rt, 12 h, (yields 95–100%) (*iv*) morpholine, MeCN, 140 °C, MW, 7 h, (yield 65%).

Subsequent heating of 2,4-dichloroquinolines **5**–**7** with respective benzylamines in DMSO afforded aromatic amines **8a**–**8f** (Scheme 2). Although nucleophilic displacement of chlorine at the 4 position is accompanied by the formation of a 2-substituted regioisomer, the two products were easily separated by column chromatography. Subsequent substitution with Boc-piperazine (compounds **8a**–**8f**) or morpholine (**8c**) was performed in acetonitrile upon microwave-assisted conditions. Finally, treatment of Boc-protected amines with a solution of hydrochloric acid in isopropanol yielded piperazine derivatives **9a**–**9f** as hydrochloride salts. Morpholine derivative **10** was obtained as a free base.

### 2.2. Anti-H. pylori Activity of Compounds ***9a***–***9f*** and ***10***

The antibacterial activity of obtained compounds **9a**–**9f** and **10** was assessed by the broth microdilution method [15,16] (Figure S1-SI) using a set of *cag*A+ reference and clinical *H. pylori* strains, which display various susceptibility profiles (Table 1).

Metronidazole was applied as a positive control [17]. Compounds were regarded as effective anti-*H. pylori* agents if their MIC values did not exceed 4 µg/mL [18,19]. The initially identified compound **9a** with low inhibitory activity against metronidazole-resistant strain (ATCC 43504) also displayed low activity against the CagA-positive susceptible strain (J99) clarithromycin-resistant strain (ATCC 700684) and CagA-positive multidrug-resistant strain (3CML) (Table 1).

Compounds bearing chlorine atoms at *orto* (**9b**) or *para* (**9d**) position of the phenyl ring exhibited greater activity against metronidazole-resistant (ATCC 43504) and CagA positive susceptible (J99) strains than compound **9a**. Notably, the *meta*-substituted congener (**9c**) preserved its *anti-H. pylori* activity against the metronidazole-resistant strain and exhibited superior inhibitory efficacy against other tested strains.

Compound **9c** demonstrated strong inhibitory activity against the *H. pylori* J99 strain. This strain contains oncoprotein CagA, which is regarded as one of the most important virulence factors, which may induce malignant neoplasms in mammals. Considering the favorable effect of chlorine at the *meta* position of the phenyl ring, further structural modifications were made in amine and quinoline fragments. Morpholine derivative (**10**) showed decreased inhibitory potency when compared with the piperazine analog **9c**, indicating the importance of the basic nitrogen atom for antibacterial activity. Compounds **9e** and **9f** substituted with methoxy groups at the 7 or 8 position of the quinoline system displayed moderate to low activity against *H. pylori* strains.

### 2.3. Selectivity of Compound ***9c*** over Mammalian Cells

The preliminary safety in vitro profile of compound **9c** was assessed over mammalian cells, i.e., sheep red blood cells and reference human cell lines (keratinocytes HaCaT) as well as fibroblasts derived from gastric mucosa of the patient.

Compound **9c** exhibited no hemolytic activity (lysis < 3%) against sheep red blood cells at concentrations up to 200 µM when compared to positive control Triton-X 100. The cellular viability of HaCaT was determined by an MTT assay, which evaluates mitochondrial enzyme activity. Compound **9c** displayed IC_50_ values of 5 μg/mL (corresponding 12 µM).

Further testing on human gastric fibroblasts using trypan blue staining and green/red fluorescent staining showed that compound **9c** at doses 1.5–3 µg/mL produced no reduction in cell viability, though there was reduced cell viability at 4 µg/mL (*p* < 0.05) [detailed description in SI p. 7].

### 2.4. Metabolic Stability of Compound ***9c***

Metabolic stability refers to the susceptibility of compounds to biotransformation, which impacts their efficacy and safety. Compound **9c** was evaluated in vitro metabolic stability assays using rat liver microsomes [20]. Compound **9c** demonstrated metabolic stability, with 85% of the compound remaining in the reaction mixture after 30 min of incubation and exhibited a low clearance value (Cl_int_ = 12.2 µL/min/mg).

### 2.5. Bacteriostatic Activity of Compound ***9c***

Assessment of the bactericidal/bacteriostatic activity of compound **9c** was carried out against metronidazole and clarithromycin-resistant *H. pylori* strains (ATCC 43504 and ATCC 700684, respectively) as well as in the *cag*A+ susceptible strain (Table 2). The MBC/MIC ratio for compound **9c** exceeds 4, which indicates bacteriostatic activity and therefore its capacity to inhibit the growth of microorganisms.

### 2.6. Anti-Biofilm Activity of Compound ***9c***

**Figure 3:**
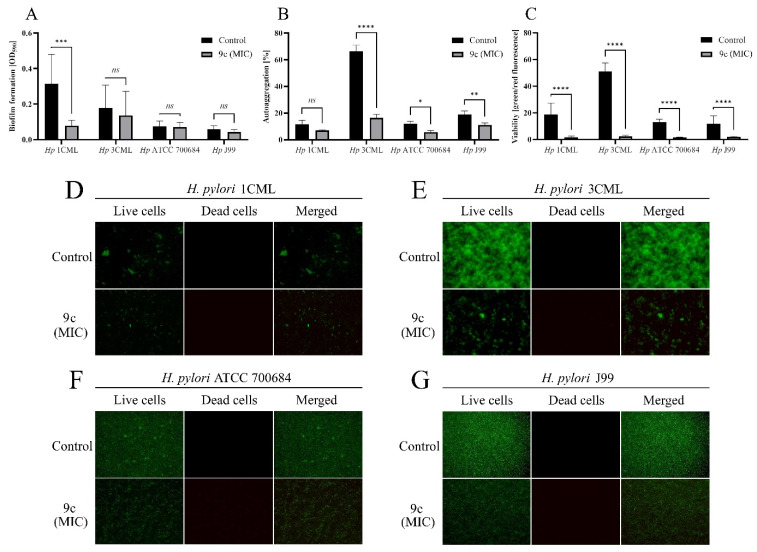
Anti-biofilm activity of compound **9c** against clinical and reference *H. pylori* strains. (**A**) Reduction in biofilm biomass production, as determined by crystal violet staining (*n* = 6), showing a statistically significant decrease in biomass with compound **9c** treatment; (**B**) Inhibition of autoaggregation, reflected in the reduced coverage of the observation field, as measured through microscopic observations (*n* = 3); (**C**) Decrease in bacterial viability within the biofilm, as assessed by the LIVE/DEAD staining kit during microscopic observations (*n* = 27), indicating substantial loss of viable bacteria; (**D**–**G**) Representative images of fluorescently stained biofilms of four tested *H. pylori* strains showing a marked reduction in biofilm formation with **9c** treatment. *ns*, statistically insignificant; asterisks indicate statistical significance with *p* values being lower than 0.05 (*), 0.01 (**), 0.001 (***) and 0.0001 (****).

The formation of biofilm on the surface of the gastric mucosa is most likely the primary cause of long-term chronic inflammations and multiple drug resistance. As a result, anti-biofilm agents have been investigated in recent years as an alternative or complementary therapy to currently used antibiotics and chemotherapeutics. Since clinical strains isolated from the gastric mucosa of patients have been reported to have a higher capacity to form biofilm than tested reference strains, the anti-biofilm activity of compound **9c** was tested against two clinical multidrug-resistant strains: 1CML and 3CML, and compared to two reference strains: ATCC 700684 and J99 (Figure 3).

We investigated the effects of compound **9c** on *H. pylori* biofilm, including its effect on biofilm formation, bacterial viability within the biofilm, and disruption of auto-aggregation a key initial step in biofilm development. The inhibitory properties of compound **9c** on biofilm formation were evaluated by crystal violet staining followed by spectrophotometric measurements. Compound **9c** showed a strain-specific, anti-biofilm effect ranging from 10% to 75%, with the highest activity against *H. pylori* 1CML strain (75% decrease) (Figure 3).

The impact of compound **9c** on the bacterial cell viability in a structured biofilm was investigated by fluorescence microscopy after treatment with green and red fluorescent nucleic acid stains (SYTO9 and propidine iodide, respectively), which covalently label live and dead cells. In biofilms treated with **9c**, a significant reduction in the LIVE/DEAD cells (green/red) ratio was observed, i.e., from a six-fold decrease in *H. pylori* J99 to 22-fold in *H. pylori* 3CML (Figure 3).

One of the first steps in biofilm formation is auto-aggregation—the ability of bacteria cells for self-binding. As revealed by fluorescent microscopy observations, treatment with compound **9c** resulted in a four-fold decline of the auto-aggregation in *H. pylori* 3CML strain, whereas, in other tested strains, a two-fold reduction was observed (Figure 3).

Thus, compound **9c** at MIC values enabled the destruction of biofilm produced by *H. pylori* via eradication of a structured biofilm, prevention of the auto-aggregation of microbial cells and inhibition of the biofilm formation.

### 2.7. Evaluation of Antibacterial Spectrum of Compound ***9c***

The use of drugs that affect the gut microbiota is associated with an increased risk of bacterial infections that can negatively affect the microbiota and lead to patient deterioration [21]. Given the long-term nature of *H. pylori* treatment, we evaluated the antibacterial spectrum of compound **9c** to determine its selectivity and potential therapeutic advantage. This compound is selectively active only against *H. pylori*, safe for the microbiota, and increases the safety of long-term treatment.

The selective antibacterial activity of potential chemotherapeutics against *H. pylori* with minimal impact on other bacteria, particularly gut microbiota, is of particular importance. Thus, we assessed the selectivity of compound **9c** against *H. pylori* and found that it showed no activity against the reference strains of *S. aureus*, *E. coli* or *E. faecalis*. Moreover, **9c** did not exhibit any inhibitory effects on *L. paracasei*, a key component of the gut microbiota (Table 3). According to EUCAST harmonization guidelines, gentamicin was used as a control drug for all tested strains to ensure the quality of MIC determination [17].

### 2.8. Synergistic Effects of Compound ***9c***

The increasing drug resistance of bacterial strains together with a narrow panel of antibiotics and chemotherapeutics that are used to treat *H. pylori* infections urges the search for new compounds that will not only exhibit antibacterial effects against *H. pylori* but also enhance the activity of other drugs. Synergistic therapies have proven to be the most effective strategy to overcome bacterial resistance [22]. The interaction between tested compounds is determined by the fractional inhibitory concentration (FIC) index. Values lower than 0.5 are assumed to be synergistic, results ranging from 0.5–1.0 give additivity (partial synergism) values higher than 1.0, but those lower than 4.0 show neutral interactions, while a FICI of >4.0 indicates antagonism [23].

A joint treatment of compound **9c** (at MIC = 4 µg/mL and 1/2 × MIC = 2 µg/mL) and clarithromycin or metronidazole against ATCC 700684 and 3CML *H. pylori* strain reduced the MIC values of the latter up to 16-fold and 64-fold, respectively (Table 4). Moreover, the administration of compound **9c** and metronidazole (at MIC = 4 µg/mL and 1/2 × MIC = 2 µg/mL) reduced the MIC values for the 3CML strain. Of note, the case of administration of compound **9c** at its MIC value resulted in the restoration of the strain’s susceptibility to metronidazole, despite the strain being initially resistant (Table 4).

Compound **9c** administered at 1/2 × MIC in combination with clarithromycin revealed an additive effect for both *H. pylori* ATCC 700684 and 3CML strains, as shown by FIC values of 0.75 (Table 5 and Table 6).

Similarly, the additive effect between compound **9c** (at 1/2 × MIC) and metronidazole was observed against the 3CML strain (Table 6). Thus, the additive activity presented by **9c** might be regarded as a potential strategy to enhance the antibacterial activity of antibiotics and chemotherapeutics applied in the treatment of resistant *H. pylori* strains.

### 2.9. Activity of Compound ***9c*** Against Human Gastric Cancer Cell Line SNU-1

**Figure 4:**
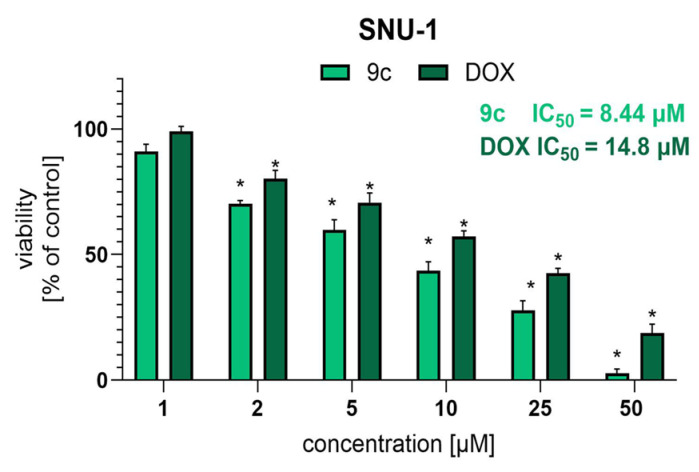
The effect of compound 9c on the viability of human gastric cancer SNU-1 cells after 24 h incubation. Cells were seeded at a density of 1 × 10^4^ cell/well, then compounds **9c** or doxorubicin (DOX) were added on concentrations of 1–50 µM. Next, an MTT assay was performed to assess the metabolic activity of cells. The Graph shows the mean viability ± SD, *n* = 6. * *p* < 0.05, statistical significance was determined using the Brown–Forsythe and Welch’s ANOVA tests, along with the post-hoc unpaired *t*-test with Welch’s correction.

Given the increased risk of gastric cancer associated with CagA-positive *H. pylori* infections, the anticancer potential of compound **9c** was evaluated against the human gastric cell line (SNU-1). The viability of cancer cells was measured using the MTT assay, which assesses mitochondrial enzyme activity. Under carefully controlled experimental conditions, this activity correlates with cell viability. Compound **9c** displayed a cytotoxic effect after 24 h incubation with IC_50_ equaling 8.44 µM (corresponding to 3.28 µg/mL) [Figure 4].

## 3. Materials and Methods

### 3.1. Chemistry

#### 3.1.1. Synthesis General Information

The synthesis was conducted at room temperature, unless indicated otherwise. Organic solvents (from Sigma-Aldrich, St. Louis, MO, USA and Prochem, Konstancin-Jeziorna, Poland) were of reagent grade and were used without purification. All reagents (Merck, Darmstadt, Germany; Fluorochem, Hadfield, UK; and TCI, Tokyo, Japan.) were of the highest purity. Column chromatography was performed on silica gel Merck 60 (70–230 mesh ASTM).

The UPLC analyses and high-resolution mass spectra (LC-HRMS) were obtained on Waters ACQUITY I-Class PLUS™ SYNAPT XS High-Resolution Mass Spectrometer (Waters, Milford, CT, USA) with the MS-Q-TOF detector and UV-Vis-DAD eλ detector. The ACQUITY UPLC BEH C18, 1.7 μm (2.1 × 100 mm) column was used with the VanGuard Acquity UPLC BEH C18, 1.7 μm (2.1 × 5 mm) (Waters, Milford, CT, USA). Standard solutions (1 mg/mL) of each compound were prepared in analytical grade MeCN/water mixture (1:1; *v*/*v*). Conditions applied were as follows: eluent A (water/0.1% HCOOH), eluent B (MeCN/0.1% HCOOH), a flow rate of 0.3 mL/min, a gradient of 5–100% B over 13 min, and an injection volume of 1 μL. The UPLC/MS purity of all the test compounds and key intermediates was determined to be >95%.

^1^H NMR and ^13^C NMR spectra were recorded using JEOL JNM-ECZR 500 RS1 (ECZR version; JEOL-Ltd., Tokyo, Japan) at 500 and 126 MHz, respectively, and are reported in ppm using deuterated solvent for calibration (CDCl_3_, methanol-*d*_4_ or dmso-*d*_6_). The *J* values are given in Hertz (Hz). Melting points were determined with the Büchi apparatus and are uncorrected.

#### 3.1.2. General Procedure for Preparation of Compounds **3** and **4**

Respective methoxyquinoline (1 g, 5.2 mmol, 1 eq) was dissolved in DCM (15 mL). mPCBA (1.4 g, 8 mmol, 1.5 eq) was added and the reaction mixture was stirred at room temperature for 30 min. The solvent was evaporated, and the product was isolated using column chromatography with DCM/MeOH 9/0.7 as a developing solvent.

*4-Chloro-7-methoxyquinolin-2(1H)-one* (**3**)

Beige solid, yield 85%, *t*_R_ = 4.96 min, Mp 250–252 °C, C_10_H_8_ClNO_2_, MW 209.63. ^1^H NMR (500 MHz, dmso-*d*_6_) δ ppm 3.84 (s, 3H), 6.62 (s, 1 H), 6.86 (d, *J* = 2.6 Hz, 1 H), 6.91 (m, 1 H), 7.77 (d, *J* = 9.1 Hz, 1 H), 8.53 (d, *J* = 6.7 Hz, 1 H). ^13^C NMR (126 MHz, dmso-*d*_6_) δ ppm 56.6, 99.3, 119.4, 122.8, 127.0, 128.3, 129.3, 136.4, 160.4, 167.4. Monoisotopic mass 209.01, [M + H]^+^ = 210.1. Spectral data are in accordance with the literature [14].

*4-Chloro-8-methoxyquinolin-2(1H)-one* (**4**)

Beige solid, yield 80%, *t*_R_ = 4.51 min, Mp 167–168 °C, C_10_H_8_ClNO_2_, MW 209.63. ^1^H NMR (500 MHz, dmso-*d*_6_) δ ppm 3.97 (s, 3 H), 7.45–7.54 (m, 1 H), 7.80 (s, 1 H), 8.12 (d, *J* = 9.0 Hz, 1 H), 8.62 (d, *J* = 6.5 Hz, 1 H). ^13^C NMR (126 MHz, dmso-*d*_6_) δ ppm 56.8, 112.8, 115.3, 122.3, 127.5, 131.0, 133.9, 160.5, 166.6. Monoisotopic mass 209.01, [M + H]^+^ = 210.1.

#### 3.1.3. General Procedure for Preparation of Compounds **5** and **6**

The respective compound **3** or **4** (800 mg, 3.8 mmol, 1 eq) was dissolved in POCl_3_ (15 mL) and the mixture was stirred at 105 °C for 1 h. The mixture was poured on crushed ice and the appearing precipitate was filtered and dried under vacuum.

*2,4-Dichloro-7-methoxyquinoline* (**5**)

Beige solid, yield 65%, *t*_R_ = 8.85 min, Mp 120–123 °C, C_10_HCl_2_NO, MW 228.07. ^1^H NMR (500 MHz, dmso-*d*_6_) δ ppm 3.95 (s, 3 H), 7.39 (d, *J* = 2.4 Hz, 1 H), 7.45 (m, 1 H), 7.73 (s, 1 H), 8.07 (d, *J* = 8.9 Hz, 1 H). ^13^C NMR (126 MHz, dmso-*d*_6_) δ ppm 56.5, 112.4, 115.3, 124.2, 126.7, 130.5, 140.2, 143.9, 148.2, 155.1. Monoisotopic mass 226.99, [M + H]^+^ = 228.0. Spectral data are in accordance with the literature [14].

*2,4-Dichloro-8-methoxyquinoline* (**6**)

White solid, yield 70%, *t*_R_ = 7.82 min, Mp 137–139 °C, C_10_H_7_Cl_2_NO, MW 228.07. ^1^H NMR (500 MHz, dmso-*d*_6_) δ ppm 3.93 (s, 3 H), 7.14 (d, *J* = 7.9 Hz, 1 H), 7.52–7.59 (m, 2 H), 7.75 (d, *J* = 8.5 Hz, 1 H). ^13^C NMR (126 MHz, dmso-*d*_6_) δ ppm 56.6, 111.4, 115.4, 123.0, 126.2, 129.6, 139.5, 143.9, 148.2, 155.1. Monoisotopic mass 226.99, [M + H]^+^ = 228.0.

#### 3.1.4. General Procedure for Preparation of Compounds **8a**–**8d**

2,4-dichloroquinoline **7** (300 mg, 1.5 mmol, 1 eq) was dissolved in DMSO, followed by the addition of respective benzylamine (2.25 mmol, 1.5 eq). The mixture was stirred at 140 °C for 3 h upon microwave irradiation. The resulting solution was diluted with AcOEt, washed three times with water, once with brine, dried over Na_2_SO_4_ and evaporated. The obtained crude product was purified using column chromatography with DCM/MeOH 9/0.5 as a developing solvent.

*2-Chloro-N-(3-methylbenzyl)quinolin-4-amine* (**8a**)

Pale oil, yield 35%, *t*_R_ = 5.40 min, C_17_H_15_ClN_2_, MW 382.8, ^1^H NMR (500 MHz, CDCl_3_) δ ppm 2.34 (s, 3 H), 4.64 (s, 2H), 5.21 (s, 1 H), 6.76 (s, 1 H), 7.09 (d, *J* = 7.2 Hz, 1 H), 7.14–7.25 (m, 3 H), 7.29 (d, *J* = 8.2, Hz, 1 H), 7.58 (d, *J* = 8.4 Hz, 1 H), 7.67–7.72 (m, 1 H), 7.98 (d, *J* = 8.2 Hz, 1 H). ^13^C NMR (126 MHz, CDCl_3_) δ ppm 21.4, 45.9, 111.0, 121.8, 123.0, 124.15, 124.9, 126.6, 128.3, 130.7, 138.5, 138.8, 143.1, 148.7, 156.3. Monoisotopic mass 282.09, [M + H]^+^ = 283.3.

*2-Chloro-N-(2-chlorobenzyl)quinolin-4-amine* (**8b**)

Pale oil, yield 42%, *t*_R_ = 6.08 min, C_16_H_12_Cl_2_N_2_, MW 303.2, ^1^H NMR (500 MHz, CDCl_3_) δ ppm 4.80 (s, 2 H), 5.24 (s, 1 H), 6.76 (s, 1 H), 7.19–7.23 (m, 2 H), 7.29 (d, *J* = 8.2 Hz, 1 H), 7.36–7.39 (m, 1 H), 7.49–7.52 (m, 1 H), 7.57 (d, *J* = 8.4, 1 H), 7.70 (d, *J* = 7.9 Hz, 1 H), 7.98 (d, *J* = 8.2 Hz, 1 H). ^13^C NMR (126 MHz, CDCl_3_) δ ppm 43.5, 108.3, 111.0, 121.8, 123.1, 124.2, 126.6, 127.0, 128.8, 130.7, 133.7, 136.4, 143.2, 148.5, 149.2, 156.0. Monoisotopic mass 302.04, [M + H]^+^ = 303.1.

*2-Chloro-N-(3-chlorobenzyl)quinolin-4-amine* (**8c**)

Pale oil, yield 45%, *t*_R_ = 6.11 min, C_16_H_12_Cl_2_N_2_, MW 303.2, ^1^H NMR (500 MHz, CDCl_3_) δ ppm 4.68 (s, 2 H), 5.14 (s, 1 H), 6.74 (s, 1 H), 7.22–7.27 (m, 3 H), 7.28–7.33 (m, 1 H), 7.37 (s, 1 H), 7.55–7.60 (m, 1 H), 7.67–7.71 (m, 1 H), 7.97–8.01 (m, 1 H). ^13^C NMR (126 MHz, CDCl_3_) δ ppm 45.2, 111.1, 121.9, 123.2, 124.2, 125.8, 127.6, 127.8, 130.0, 130.8, 134.5, 141.3, 143.23, 148.6, 156.00. Monoisotopic mass 302.04, [M + H]^+^ = 303.2.

*2-Chloro-N-(4-chlorobenzyl)quinolin-4-amine* (**8d**)

Pale oil, yield 40%, *t*_R_ = 5.99 min, C_16_H_12_Cl_2_N_2_, MW 303.2, ^1^H NMR (500 MHz, CDCl_3_) δ ppm 4.71 (s, 2 H), 5.15 (s, 1 H), 6.75 (s, 1 H), 7.15–7.27 (m, 2 H), 7.30 (d, *J* = 8.2 Hz, 1 H), 7.42 (d, *J* = 7.1 Hz, 2 H), 7.57 (d, *J* = 8.4, 1 H), 7.63 (d, *J* = 7.2 Hz, 2 H). ^13^C NMR (126 MHz, CDCl_3_) δ ppm 45.1, 111.0, 121.8, 123.3, 124.2, 126.4, 128.9, 129.1, 130.9, 133.3, 137.5, 143.4, 148.3, 156.0. Monoisotopic mass 302.04, [M + H]^+^ = 303.1.

#### 3.1.5. General Procedure for Preparation of Compounds **8e** and **8f**

Compounds **8e** and **8f** were synthesized according to the general procedure used for the preparation of compounds **8a**–**8d**, with compounds **5** and **6** serving as substrates in place of compound **7**.

*2-Chloro-N-(3-chlorobenzyl)-7-methoxyquinolin-4-amine* (**8e**)

Pale oil, yield 30%, *t*_R_ = 6.76 min, C_17_H_14_Cl_2_N_2_O, MW 333.21. ^1^H NMR (500 MHz, CDCl_3_) δ ppm 3.90 (s, 3 H), 4.67 (d, *J* = 5.6 Hz, 2 H), 6.60 (s, 1 H), 6.95 (dd, *J* = 9.0, 2.6 Hz, 1 H), 7.07 (d, *J* = 2.6 Hz, 1 H), 7.21–7.28 (m, 3 H), 7.37 (s, 1 H), 7.87 (d, *J* = 9.0 Hz, 1 H). ^13^C NMR (126 MHz, CDCl_3_) δ ppm 47.0, 55.6, 97.9, 107.8, 112.0, 117.6, 120.9, 125.6, 127.6, 128.3, 130.4, 135.0, 139.1, 149.7, 151.4, 152.3, 161.3. Monoisotopic mass 332.05, [M + H]^+^ = 333.2

*2-Chloro-N-(3-chlorobenzyl)-8-methoxyquinolin-4-amine* (**8f**)

Pale oil, yield 42%, *t*_R_ = 6.96 min, C_17_H_14_Cl_2_N_2_O, MW 333.21. ^1^H NMR (500 MHz, CDCl_3_) δ ppm 4.02 (s, 3 H), 4.52 (d, *J* = 5.44 Hz, 2 H), 6.42 (s, 1 H), 7.04–7.07 (m, 1 H), 7.23–7.26 (m, 1 H), 7.25 (s, 1 H), 7.29–7.32 (m, 2 H), 7.35–7.39 (m, 1 H), 7.39–7.44 (m, 2 H). ^13^C NMR (126 MHz, CDCl_3_) δ ppm 49.09, 56.05, 99.87, 109.03, 111.46, 118.72, 125.66, 127.58, 128.26, 130.37, 134.98, 139.01, 150.98, 151.45, 154.93. Monoisotopic mass 332.05, [M + H]^+^ = 333.2.

#### 3.1.6. General Procedure for Preparation of Compounds **9a**–**9f** and **10**

Respective benzylamine derivative **8a**–**8f** (0.35 mmol, 1 eq) was suspended in MeCN followed by the addition of TEA (1 mmol, 3 eq) and Boc-piperazine (**9a**–**9f**) or morpholine (**10**) (1.4 mmol, 4 eq). The mixture was stirred at 140 °C for 7 h upon microwave irradiation. The solvent was evaporated and the remaining crude product was purified using column chromatography with AcOEt/Hex as a developing solvent. Compounds **9a**–**9f** were subsequently suspended in ethanol and 6N HCl in isopropanol was added. After completion of the reaction, the obtained hydrochloride salt was filtered and dried under vacuum.

*N-(3-methylbenzyl)-2-(piperazin-1-yl)quinolin-4-amine hydrochloride* (**9a**)

White solid, yield 50%, *t*_R_ = 3.67 min, Mp 145–147 °C, C_20_H_24_N_4_·HCl, MW 368.9, ^1^H NMR (500 MHz, methanol-*d*_4_) δ ppm 2.32–2.37 (s, 3 H), 3.50–3.63 (m, 8 H), 4.71 (s, 2 H), 6.53 (s, 1 H), 7.16 (d, *J* = 7.5 Hz, 1 H), 7.19–7.23 (m, 1 H), 7.26 (s, 1 H), 7.27–7.32 (m, 1 H), 7.48–7.54 (m, 1 H), 7.74 (d, *J* = 8.3, 1 H), 7.79–7.87 (m, 1 H), 7.96 (d, *J* = 8.2 Hz, 1 H). ^13^C NMR (126 MHz, methanol-*d*_4_) δ ppm 20.1, 43.1, 45.9, 118.1, 123.5, 124.9, 128.3, 128.7, 132.4, 137.7, 138.7, 153.4. Monoisotopic mass 332.20, [M + H]^+^ = 333.3.

*N-(2-chlorobenzyl)-2-(piperazin-1-yl)quinolin-4-amine hydrochloride (***9b**)

White solid, yield 45%, *t*_R_ = 3.55 min, Mp 188–190 °C, C_20_H_21_ClN_4_·HCl, MW 389.3. ^1^H NMR (500 MHz, methanol-*d*_4_) δ ppm 3.50–3.63 (m, 8 H), 4.86 (s, 2 H), 6.53 (s, 1 H), 7.32–7.41 (m, 2 H), 7.46–7.55 (m, 3 H), 7.71–7.78 (m, 1 H), 7.79–7.88 (m, 1 H), 7.98 (d, *J* = 8.2 Hz, 1 H). ^13^C NMR (126 MHz, methanol-*d*_4_) δ ppm 43.2, 44.2, 118.2, 125.1, 127.5, 129.2, 132.5, 133.7, 137.7, 153.6. Monoisotopic mass 352.15, [M + H]^+^ = 353.2.

*N-(3-chlorobenzyl)-2-(piperazin-1-yl)quinolin-4-amine hydrochloride* (**9c**)

White solid, yield 55%, *t*_R_ = 3.70 min, Mp 155–157 °C, C_20_H_21_ClN_4_·HCl, MW 389.3. ^1^H NMR (500 MHz, methanol-*d*_4_) δ ppm 3.50–3.64 (m, 8 H), 4.79 (s, 2 H), 6.55 (s, 1 H), 7.31–7.35 (m, 1 H), 7.39 (d, *J* = 5.30 Hz, 2 H), 7.46–7.55 (m, 2 H), 7.74 (t, *J* = 7.73 Hz, 1 H), 7.83 (d, *J* = 0.72 Hz, 1 H), 7.97 (d, *J* = 7.73 Hz, 1 H). ^13^C NMR (126 MHz, methanol-*d*_4_) δ ppm 43.1, 45.2, 118.1, 124.9, 125.0, 126.0, 130.3, 134.5, 153.5. Monoisotopic mass 352.15, [M + H]^+^ = 353.1. HRMS calcd for C_20_H_22_ClN_4_, 353.1533; found 353.1517.

*N-(4-chlorobenzyl)-2-(piperazin-1-yl)quinolin-4-amine hydrochloride* (**9d**)

White solid, yield 47%, *t*_R_ = 3.80 min, Mp 112–115 °C, C_20_H_21_ClN_4_·HCl, MW 389.3. ^1^H NMR (500 MHz, methanol-*d*_4_) δ ppm 3.47–3.63 (m, 8 H), 4.76 (s, 2 H), 6.48–6.58 (m, 1 H), 7.33–7.46 (m, 1 H), 7.38–7.42 (m, 1 H), 7.39–7.45 (m, 1 H), 7.42–7.45 (m, 1 H), 7.47–7.57 (m, 1 H), 7.75 (t, *J* = 7.8 Hz, 1 H), 7.82 (s, 1 H), 7.97 (d, *J* = 8.0 Hz, 1 H). ^13^C NMR (126 MHz, methanol-*d*_4_) δ ppm 43.2, 45.2, 118.2, 125.1, 127.6, 132.5, 134.7, 137.7, 153.5. Monoisotopic mass 352.15, [M + H]^+^ = 353.1.

*N-(3-chlorobenzyl)-7-methoxy-2-(piperazin-1-yl)quinolin-4-amine hydrochloride* (**9e**)

White solid, yield 65%, t*_R_* = 3.99 min, Mp 220–222 °C, C_21_H_23_ClN_4_O·HCl, MW 419.35. ^1^H NMR (500 MHz, methanol-*d*_4_) δ ppm 3.46–3.63 (m, 8 H), 3.80–3.99 (s, 3 H), 4.77 (s, 2 H), 6.38 (bs, 1 H), 7.02–7.16 (m, 1 H), 7.30–7.36 (m, 4 H), 7.47 (s, 1 H), 7.85 (d, *J* = 9.02 Hz, 1 H). ^13^C NMR (126 MHz, methanol-*d*_4_) δ ppm 43.2, 45.1, 55.2, 114.8, 125.9, 126.5, 127.5, 128.0, 130.3, 134.5, 139.8, 153.7, 163.3. Monoisotopic mass 382.16, [M + H]^+^ 383.3.

*N-(3-chlorobenzyl)-8-methoxy-2-(piperazin-1-yl)quinolin-4-amine hydrochloride* (**9f**)

White solid, yield 41%, t*_R_* = 5.15 min, Mp 204–206 °C, C_21_H_23_ClN_4_O·HCl, MW 419.35. ^1^H NMR (500 MHz, methanol-*d*_4_) δ ppm 3.41 (br s, 4 H), 3.80–3.89 (m, 4 H), 4.07 (s, 3 H), 4.79 (s, 2 H), 6.01 (s, 1 H), 7.24–7.30 (m, 1 H), 7.32–7.42 (m, 3 H), 7.43–7.52 (m, 2 H), 7.81 (d, *J* = 8.31 Hz, 1 H). ^13^C NMR (126 MHz, methanol-*d*_4_) δ ppm 42.7, 44.5, 45.7, 56.0, 84.8, 112.4, 113.1, 115.3, 125.6, 127.1, 127.6, 130.3, 134.5, 139.2, 148.5, 153.5, 155.6. Monoisotopic mass 382.16, [M + H]^+^ 383.2.

*N-(3-chlorobenzyl)-2-morpholinoquinolin-4-amine* (**10**)

White solid, yield 67%, *t*_R_ = 5.67 min, Mp 115–116 °C, C_20_H_20_ClN_3_O, MW 353.86. ^1^H NMR (500 MHz, methanol-*d*_4_) δ ppm 3.07–3.12 (m, 4 H), 3.88–3.92 (m, 4 H), 4.63 (s, 2 H), 6.28 (s, 1 H), 7.15 (d, *J* = 8.2 Hz, 1 H), 7.19–7.23 (m, 1 H), 7.24–7.31 (m, 2 H), 7.39 (t, *J* = 1.4 Hz, 1 H), 7.43 (dd, *J* = 8.4, 6.9 Hz, 1 H), 7.57 (d, *J* = 8.5 Hz, 1 H), 7.81 (d, *J* = 8.3 Hz, 1 H). ^13^C NMR (126 MHz, dmso-*d*_6_) δ ppm 28.6, 43.8, 79.6, 100.4, 119.3, 121.2, 123.8, 127.0, 127.8, 129.4, 130.6, 133.4, 143.9, 154.5, 156.8, 157.8. Monoisotopic mass 353.13, [M + H]^+^ 354.2.

### 3.2. Antibacterial Activity of Tested Compounds Against H. pylori

#### 3.2.1. *H. pylori* Strains

The set of *H. pylori* strains studied includes 4 strains with different susceptibility patterns, three reference strains and four clinical strains (Table S1-SI). Reference strains were derived from the American Type Culture Collection (ATCC), while multi-drug-resistant (MDR) clinical strains were derived from the culture collection of the Department of Microbiology, Wroclaw Medical University. Strains were stored at −80 °C as microbank and subsequently thawed at room temperature. Then, suspensions were inoculated onto Schaedler agar with 5% sheep blood (bioMerieux, Marcy-l’Étoile, France) and incubated for 72 h at 37 °C under microaerophilic conditions. After the incubation, pure cultures were used for further analysis.

#### 3.2.2. Evaluation of MIC Values of Tested Compounds

The minimum inhibitory concentration (MIC) of seven compounds (**9a**–**9f** and **10**) and quipazine against four *H. pylori* strains (three references and one clinical MDR—CML 3) was assessed by microdilution in brain heart infusion broth (BHI Broth, Oxoid) supplemented with 5% fetal bovine serum (FBS, Sigma-Aldrich, St. Louis, MO, USA), according to the CLSI recommendations [25].

Briefly, two-fold serial dilution of tested compounds ranging from 16 µg/mL to 0.5 µg/mL were prepared in 24-well flat bottom titrate plates (Sarstedt, Nümbrecht, Germany). Each tested well was inoculated with bacterial suspension to obtain the final volume of 1 mL and bacterial concentration of 10^6^ colony-forming unites (CFU)/mL Plates were incubated with shaking (140 rpm) for 72 h at 37 °C under microaerophilic conditions (GENbag Microaer, bioMerieux, Marcy-l’Étoile, France). After the incubation, the MIC values were evaluated as the lowest concentration that inhibits bacterial growth. The MIC was determined by measuring the optical density at 600 nm (OD_600_) using a Sunrise plate reader (Tecan, Männedorf, Switzerland).

Tested compounds were dissolved in DMSO (<2%*v*/*v*) and did not reveal any impact on *H. pylori* growth in BHI broth. Susceptibility to reference chemotherapeutic (metronidazol), used as a control was evaluated in accordance with EUCAST clinical breakpoints tables [17]. The tests were run in triplicate.

The antibacterial activity of the newly synthesized compounds was considered significant when the MIC values were equal to or smaller than 4 µg/mL.

### 3.3. Assessment of Selectivity of Compound ***9c*** over Mammalian Cells

#### 3.3.1. Haemolytic Activity Assay

Defibrinated sheep blood (Biomaxima, Lublin, Poland) was centrifuged (3000 rpm, 10 min at 4 °C) and washed with PBS for the three times. The cell pellet was then resuspended in PBS to obtain an 8% suspension of red cells. Then, 100 μL of the resulting suspensions was treated with 100 μL of solutions of tested compounds in PBS, and they were incubated at 37 °C for 1 h. Triton X-100 (2%) and PBS were used as positive and vehicle controls, respectively. After incubation, samples were centrifugated (3000 rpm, 3 min) and 100 μL of supernatants were transferred to a 96-well microtiter plate. The haemoglobin release was determined in a SpectraMax iD3 reader (Molecular Devices, San Jose, CA, USA) by absorption at 540 nm [26,27].

#### 3.3.2. Cell Cultures

Human keratinocytes HaCaT (300493, Cytion, Eppelheim, Germany) growth in DMEM supplemented with 10% fetal bovine serum, 50 μg/mL gentamycin sulfate, pen/strep in standard culture conditions (5% CO_2_, 37 °C, 95% humidity). Cell morphology was examined using an inverted bright-field microscope, Leica DMi1 (20× objective, Leica Microsystems GmbH, Wetzlar, Germany).

#### 3.3.3. MTT Assay

Cells were seeded into 96-well plates at a density of 1 × 10^4^ cells per well. After 24 h, the cells were treated with either **9c** or metronidazole. Following a 24-h incubation, cell viability was assessed using the MTT assay. Briefly, MTT solution was added to the culture medium, and after 3 h, when the formazan crystals had formed, the medium was removed. DMSO was then added to dissolve the crystals, and absorbance was measured at 570 nm using a SpectraMax iD3 plate reader (Molecular Devices, San Jose, CA, USA). The percentage of cell viability was calculated by dividing the absorbance of the experimental condition by that of the control and multiplying it by 100%. The test was performed in triplicate.

#### 3.3.4. Impact on Human Gastric Fibroblasts

##### Fibroblast Cultures

Normal, human gastric fibroblasts: Secondary normal fibroblast cultures originating from the biopsies of patients without systemic inflammatory and autoimmunologic diseases and *Hp* infection, qualified to laparoscopic, sleeve gastrectomy were used. Fibroblasts were cultured in 5 mL of DMEM (Sigma-Aldrich, Saint Louis, MO, USA) containing 10% FBS (Sigma-Aldrich, Saint Louis, MO, USA) and antibiotics. The flasks were maintained in a humidified atmosphere of 5% CO_2_ at 37 °C, and the medium was changed every 2 days.

##### Fibroblast Viability

For viability assays, fibroblasts were seeded onto 12-well plates at the density of 1 × 10^4^ fibroblasts/well and incubated with the growing doses of **9c** (1,5, 2, 2,5, 3, 4 µg/mL) for 24 and 48 h. Fibroblasts were then harvested with trypsin and the viable and dead fibroblast number was assessed with the trypan blue (Sigma-Aldrich, Saint Louis, MO, USA) fibroblast counting in Bürker hemocytometer(Paul Marienfeld GmbH & Co. KG, Lauda-Königshofen, Germany). The viable/dead fibroblast number was additionally verified by labeling with Invitrogen^TM^ Ready Count^TM^ Green/Red Fibroblast Viability Stain (Invitrogen, Waltham, MA, USA) according to the manufacturer protocol and counting with automated fibroblast counter Countess 3 FL (Invitrogen, Waltham, MA, USA).

### 3.4. Metabolic Stability

The metabolic stability of compound **9c** was assessed following previously reported methods [20]. A solution of tested compound at a final concentration of 10 µM was pre-incubated in a phosphate buffer (pH = 7.4) containing rat liver microsomes (pooled microsomes from male rat liver, 0.5 mg/mL, Merck/Millipore Sigma, Darmstadt, Germany) for 10 min at 37 °C. Metabolic reactions were initiated by adding NADPH-regenerating system (including NADP+, glucose-6-phosphate, and glucose-6-phosphate dehydrogenase in 100 mM phosphate buffer, pH 7.4; all from Sigma Aldrich), with incubation periods of 0, 30, and 60 min at 37 °C in negative control samples, the NADPH-regenerating system was replaced with phosphate buffer. The process was stopped by the addition of an ice-cold methanol solution containing the internal standard (pentoxifylline, 100 nM). Samples were centrifuged, and the supernatant was analyzed by UPLC/MS. All tests were performed in duplicate. The half-life (t_1/2_) of the compounds was calculated from the slope of the linear regression on Ln (% parent compound remaining) versus time plots. Intrinsic clearance (Cl_int_) was calculated from the formula: Cl_int_ = [incubation volume (μL)/protein amount (mg) × 0.693]/t^1^_/2_. The assay performance was confirmed by using imipramine, an extensively metabolized drug, as a reference [28].

### 3.5. Evaluation of Bacteriostatic/Bactericidal Activity of Compound ***9c***

The effectiveness of compound **9c** to inhibit bacterial growth (bacteriostatic properties) or kill the bacteria (bactericidal properties) was evaluated against three reference *H. pylori* strains (ATCC 43504, ATCC 700684 and J99). To evaluate bacteriostatic/bactericidal activity the minimal bactericidal concentration (MBC) was evaluated, and MBC/MIC ratio was calculated following CLSI guidelines [25].

Firstly, serial dilution of **9c**, ranging from 32–0.5 µg/mL in 24-well plates, was prepared and inoculated with bacterial suspension according to the procedure previously described (Section 2.3). Then after 72-h of incubations, the 10 µL of suspension from wells with MIC, 2× MIC, 4× MIC, and 8× MIC were inoculated on Mueller Hinton Agar with horse-defibrillated blood (bioMerieux, Marcy L’Etoile, France). Then, after the incubation under microaerophilic conditions by 72 h at 37 °C, the bacterial growth was assessed. The concentration without visible bacterial colonies was found as MBC values.

A ratio of ≤4 classified the compound as bactericidal, while >4 indicated bacteriostatic properties. The MBC represents the concentration inhibiting 99.9% of bacterial growth [25].

### 3.6. Assessment of Antibiofilm Activity of Compound ***9c***

Antibiofilm activity was evaluated against four *H. pylori* strains (reference ATCC 700684 and two clinical, MDR—1CML and 3CML), using a slightly modified Christensen method [29,30].

Briefly, bacterial suspensions with a density of approximately 10^6^ CFU/mL were prepared in brain heart infusion (BHI) broth supplemented with 5% fetal bovine serum (FBS, Sigma) and were put into sterile flat-bottom, 12-well microtiter plates. Each well was filled with 1 mL of the medium containing an appropriate concentration of **9c** (referring to MIC value). Plates were incubated for 72 h at 37 °C under microaerophilic conditions with shaking at 100 rpm. After incubation, the supernatant was gently rinsed and washed with phosphate buffered saline (PBS). Then, after fixing by heating at 60 °C for 5 min, biofilms were stained by a 0.1% crystal violet solution for 15 min. Then, the dye was removed, and the bottom of each well was gently rinsed with PBS again. Subsequently, 1 mL of 96% ethanol was added to each well to dissolve the biofilm-absorbed crystal violet. The absorbance was measured at 590 nm using an Asys UVM 340 microplate reader (Biochrom Ltd., Cambridge, UK). The tests were run in triplicate.

### 3.7. Evaluation of Antibacterial Spectrum of Compound ***9c***

Compound **9c** was tested for antibacterial activity against various species of bacteria other than *H. pylori*. Gram-positive reference strains such as *Staphylococcus aureus* ATCC 29213 and *Lactobacillus paracasei* BAA 52 and Gram-negative *Escherichia coli* ATCC 25922 were used in the studies. The MIC of compound 9c was determined by the microdilution method in Mueller Hinton broth (Oxoid, Hampshire, UK). Briefly, serial two-fold dilutions of compound **9c** (50 μg/mL to 0.125 μg/mL) were prepared. A bacterial suspension (5 × 10^5^ CFU/mL) was added to each of them. After 24 h of incubation at 37 °C in aerobic conditions, the MIC was determined by measuring the optical density at 600 nm (OD_600_) using a Tecan Sunrise plate reader. All assays were performed in triplicate to ensure accuracy [15,16,17].

### 3.8. Synergistic Activity of Compound ***9c***

The synergistic activity of **9c** with metronidazole and clarithromycin the FIC values was determined using a modified checkerboard method with 24-well plates, which are optimal for *H. pylori* cultures. The activity of metronidazole and clarithromycin was assessed individually, as well as in combination with the tested compound at MIC value and 0.5× MIC, to evaluate the FIC values. The FIC index (FICI) for both agents (A—antibiotic and B—tested compound) is calculated using the following formula [24]:

ΣFIC = FIC_A + FIC_B

where:

FIC_A = MIC of agent A in combination/MIC of agent A alone

FIC_B = MIC of agent B in combination/MIC of agent B alone

### 3.9. Assessment of Cytotoxic Activity of ***9c***

#### 3.9.1. Cell Culture

Human gastric cancer cell line SNU-1 (CRL-5971, ATCC, Manassas, VA, USA) growth in RPMI supplemented with 10% fetal bovine serum, 50 µg/mL gentamycin sulfate, pen/strep in standard culture conditions (5% CO_2_, 37 °C, 95% humidity). Cell morphology was examined using an inverted bright-field microscope, Leica DMi1 (20× objective, Leica Microsystems GmbH, Wetzlar, Germany).

#### 3.9.2. Evaluation of Cytotoxicity Using MTT

Cell viability was determined by the 3-(4,5-dimethylthiazol-2-yl)-2,5-diphenyltetrazolium bromide (MTT, Sigma-Aldrich, St. Louis, MO, USA) assay. Cells were seeded into 96-well plates and cultured in the presence of **9c** compounds in a concentration range of 1–50 µM for 24 h. Doxorubicin (DOX) was used as a reference. The MTT (final concentration 0.5 mg/mL) was added to each well for 4 h and then formazan crystals were dissolved in DMSO. The absorbance was measured at 570 nm (SpectraMax^®^ iD3, Molecular Devices, San Jose, CA, USA) and the experiment was performed 3 times in duplicate.

### 3.10. Statistical Analysis

For the assessment of the impact of **9c** on human gastric fibroblasts statistical analysis of the data was performed with the use of Excel Software (Microsoft Office 365, Microsoft Corporation, Redmond, WA, USA). Each variable was expressed as the mean (±S.E.M.). The statistical significance of the difference was determined using an analysis of variance (one-way ANOVA) test (Statistica Software, version 13.3, StatSoft Inc., Tulsa, OK, USA). Further statistical analysis for post hoc comparisons was carried out with the Newman–Keuls Test. Differences were considered statistically at *p* < 0.05.

The assessment of the impact of **9c** on HaCaT and SNU-1 cell lines was determined using the Brown–Forsytheand Welch’s ANOVA tests, along with the post-hoc unpaired *t*-test with Welch’s correction.

For the analysis of anti-biofilm assays, statistical analysis was performed using GraphPad Prism version 10 (GraphPad Co., San Diego, CA, USA). The normality of the distribution was checked by the Shapiro–Wilk test. As all values were normally distributed, the two-way ANOVA test was further used. The results of statistical analyses were considered significant for values with *p* < 0.05.

## 4. Conclusions

Herein, we report on the identification of *N*-(3-chlorobenzyl)-2-(piperazin-1-yl) quinolin-4-amine, a benzylamino-quipazine derivative **9c**, which exhibited antibacterial properties against highly virulent *H. pylori* strains producing oncogenic protein CagA. Compound **9c** displayed bacteriostatic activity (MBC/MIC ratio > 4), combined with the ability to inhibit biofilm formation and prevention of bacterial cell autoaggregation. Moreover, compound **9c** in ½ MIC (2 µg/mL) showed an additive effect with clarithromycin and metronidazole, which could reduce the development of bacterial resistance. Cytotoxic activity of compound **9c** on human gastric cancer cells SNU-1 (IC_50_ = 3.28 µg/mL) suggests its importance against oncogenic properties of *H. pylori* strains. The safety profile of compound **9c**, evidenced by the absence of adverse effects on gut microbiota (*L. paracasei*) and lack of propensity to produce hemolytic activity, warrants further investigation, especially considering its unfavorable impact on the viability of mammalian cells. Finally, these results indicate that quipazine could serve as a promising core for the development of antibacterial compounds with potential applications in *H. pylori* infections and associated/related oncogenic processes.

## Figures and Tables

**Table 1:** Antibacterial activity of compounds **9a**–**9f** and **10** against selected *H. pylori* strains.

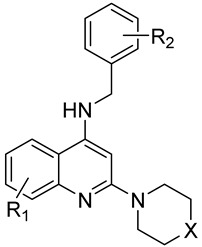								
**Cmpd**	**R_1_**	**R_2_**	**X**	**MIC [µg/mL] *^a^***				**Mean MIC** **[µg/mL]**
				**ATCC 43504 *^b^***	**ATCC 700684 *^c^***	**J99 *^d^***	**3CML *^e^***	
**9a**	H	3-CH_3_	NH	8	16	8	16	12
**9b**	H	2-Cl	NH	4	16	4	16	10
**9c**	H	3-Cl	NH	4	4	2	4	3.5
**9d**	H	4-Cl	NH	4	16	4	16	10
**9e**	7-OCH_3_	3-Cl	NH	4	16	8	16	11
**9f**	8-OCH_3_	3-Cl	NH	4	16	4	16	10
**10**	H	3-Cl	O	4	16	4	16	10
**MTZ** *^f^*				16	4	1	16	9.25
**QPZ ^g^**				16	nth	>16	nt ^h^	-

*^a^* All tests were run in triplicate, *^b^* ATCC 43504—reference, metronidazole-resistant *H. pylori* strain, *^c^* ATCC 700684—reference, clarithromycin-resistant *H. pylori* strain, *^d^* J99—reference, susceptible, *cag*A+ *H. pylori* strain, *^e^* 3CML—clinical, multi-drug-resistant *cag*A+ *H. pylori* strain, *^f^* MTZ—metronidazole, ^g^ QPZ-quipazine, ^h^ nt—not tested.

**Table 2:** Evaluation of bactericidal/bacteriostatic activity of compound **9c**.

*H. pylori* Strain	MBC [µg/mL]	MIC [µg/mL]	MBC/MIC ^*a*^ Ratio
ATCC 43504 *^b^*	>32	4	>8
ATCC 700684 *^c^*	32	4	8
J99 *^d^*	16	2	8

*^a^* MBC/MIC ratio: >4 for bacteriostatic agents, ≤4 for bactericidal agents; all tests were run in triplicate, *^b^* ATCC 43504—reference, metronidazole-resistant *H. pylori* strain, *^c^* ATCC 700684—reference, clarithromycin-resistant *H. pylori* strain, *^d^* J99—reference, susceptible, *cag*A+ *H. pylori* strain.

**Table 3:** Activity of compound **9c** against reference, susceptible *E. coli*, *S. aureus*, *E. faecalis* and *L. paracasei* bacterial strains.

Compd	MIC [µg/mL] *^a^*				
	*E. coli* ATCC 25922	*S. aureus* ATCC 25923	*S. aureus* ATCC 29213	*E. faecalis* ATCC 29212	*L. paracasei* BAA 52
**9c**	16	>32	>32	>32	32
Gentamycin	0.5	1	0.5	8	16

*^a^* All tests were run in triplicate.

**Table 4:** The impact of joint treatment of compound **9c** and clarithromycin or metronidazole against ATCC 700684 and 3CML *H. pylori* strains.

Compd	MIC [µg/mL] *^a^*	
	ATCC 700684 *^b^* (Resistant to CH)	3CML ^*c*^
**CH** *^d^*	16	16
**MTZ** *^e^*	2	128
**9c**	4	4
**CH** + **9c** (MIC)	1	2
**CH** + **9c** (1/2 × MIC)	4	4
**CH** + **9c** (1/4 × MIC)	16	16
**MTZ** + **9c** (MIC)	nt	8
**MTZ** + **9c** (1/2 × MIC)	nt	16
**MTZ** + **9c** (1/4 × MIC)	nt	64–128

*^a^* EUCAST clinical breakpoints: ≤0.25 µg/mL clarithromycin susceptible, ≥0.25 µg/mL clarithromycin-resistant, ≤8 µg/mL metronidazole susceptible, ≥8 µg/mL metronidazole-resistant [17]; All tests were run in triplicate; *^b^* ATCC 700684—reference, clarithromycin-resistant *H. pylori* strain, *^c^* 3CML—clinical, multi-drug-resistant *cag*A+ *H. pylori* strain, *^d^* CH—clarithromycin, *^e^* MTZ—metronidazole; nt—not tested.

**Table 5:** Additive effects of compound **9c** and clarithromycin on *H. pylori* ATCC 700684 strains.

Tested Compounds	ATCC 700684 *^a^*	
	FIC *^b^*	Interpretation [24]
**CH** *^c^* + **9c** (MIC)	1.0625	Indifference
**CH** + **9c** (1/2 × MIC)	0.75	Additive

*^a^* ATCC 700684—reference, clarithromycin-resistant *H. pylori* strain, *^b^* FIC—fractional inhibitory concentration *^c^* CH—clarithromycin. All tests were run in triplicate.

**Table 6:** Additive effects of compound **9c** with clarithromycin and metronidazole on *H. pylori* 3CML strain.

Tested Compounds	3CML *^a^*	
	FIC *^b^*	Interpretation [24]
**CH** *^c^* + **9c** (MIC)	0.625	Additive
**CH** + **9c** (1/2 × MIC)	0.75	Additive
**MTZ** *^d^* + **9c** (MIC)	1.0625	Indifference
**MTZ** + **9c** (1/2 × MIC)	0.625	Additive

*^a^* 3CML—clinical multi-drug-resistant *H. pylori* strain, *^b^* FIC—fractional inhibitory concentration, *^c^* CH—clarithromycin, *^d^* MTZ—metronidazole. All tests were run in triplicate.

## Data Availability

The original contributions presented in this study are included in the article and Supplementary Files.

## Supplementary Materials

The following supporting information can be downloaded at: https://www.mdpi.com/article/10.3390/ijms26135997/s1.

## Abbreviations

The following abbreviations are used in this manuscript:

BHI	Brain Heart Infusion
CH	Clarithromycin
DCM	Dichloromethane
DMSO	Dimethylsulfoxide
DOX	Doxorubicin
FBS	Fetal bovine serum
FIC	Fractional inhibitory concentration
Hex	Hexane
MBC	Minimal bactericidal concentration
MDR	Multi-drug-resistant
MIC	Minimal inhibitory concentration
mPCBA	meta-Chloroperoxybenzoic acid
MTT	3-(4,5-Dimethylthiazol-2-yl)-2,5-diphenyltetrazolium bromide
MW	Microwave
MTZ	Metronidazole
PBS	Phosphate Buffered Saline
SEM	Standard error of the mean
TEA	Triethylamine
WHO	World Health Organization
