# Supplementary material for: New Quipazine Derivatives Active Against Drug-Resistant Oncogenic Helicobacter pylori Strains with Biofilm
Source: Int J Mol Sci. 2025 Jun 22;26(13):5997. doi: 10.3390/ijms26135997 (PMC12250555; doi:10.3390/ijms26135997)
Supplement: Supplementary file 1 [file ijms-26-05997-s001.zip › ijms-3637317-supplementary.pdf]

SUPPLEMENTARY FILES

New quipazine derivatives active against drug-resistant oncogenic *Helicobacter pylori* strains with biofilm

Katarzyna Grychowska,[1*] Karolina Klesiewicz,[1*] Joanna Pęgiel,[1] Agata Kuziak,[1] Iwona Skiba-Kurek,[1] Vittorio Canale,[1] Gracjana Krzysiek-Mączka,[3] Agata Ptak-Belowska,[3] Kamil Piska,[1] Paulina Koczurkiewicz-Adamczyk,[1] Paweł Krzyżek,[2] Tomasz Brzozowski,[3] Paweł Zajdel,[1] Elżbieta Karczewska[1]

[1]*Faculty of Pharmacy, Jagiellonian University Medical College, 9 Medyczna St., 30-688 Kraków, Poland*

[2]*Department of Microbiology, Faculty of Medicine, Wrocław Medical University, 4 Chałubińskiego St., 50-368 Wroclaw, Poland*

[3] *Faculty of Medicine, Jagiellonian University Medical College, 16 Grzegórzecka Str., 31-531 Kraków, Poland*

*Corresponding authors:

karolina.klesiewicz@uj.edu.pl

katarzyna.grychowska@uj.edu.pl

Table of contents

UPLC-MS and ¹H NMR spectra of representative intermediates 8a, 8c and 8e. ... 3

UPLC-MS and ¹H NMR and ¹³ C NMR spectra of final compounds:9a-9f and 10 ... 6

Figure 1-SI. The evaluation of MIC of compounds **9c** and **9a** against reference, metronidazole resistant *H. pylori* ATCC 43504 ... 20

Impact of 9c on fibroblast viability ... 20

Figure 2-SI. Impact of **9c** on fibroblast viability. ... 22

Table 1-SI. Antimicrobial susceptibility profile of tested *H. pylori* strains. ... 23

UPLC-MS and ^{1}H NMR spectra of representative intermediates 8a, 8c and 8e.

2-Chloro-*N*-(3-methylbenzyl)quinolin-4-amine (8a)

UPLC-MS

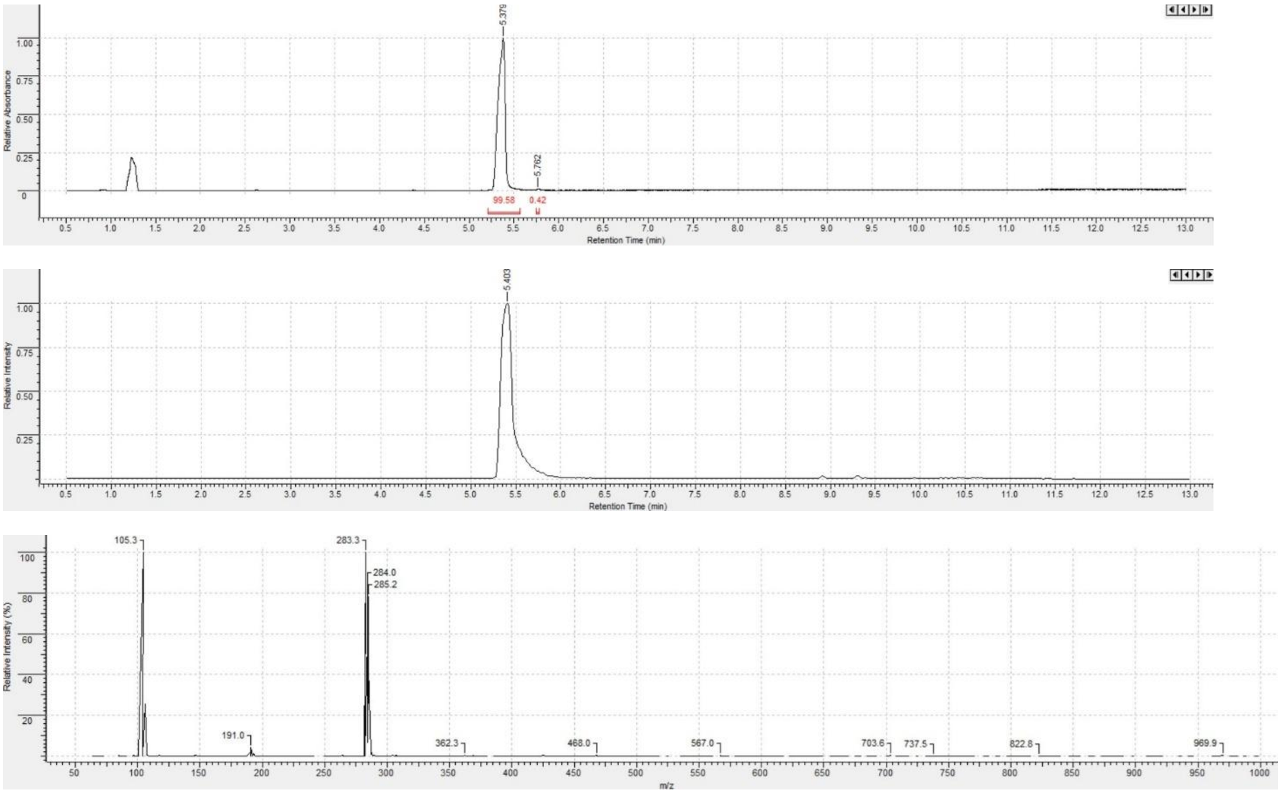

^{1}H NMR

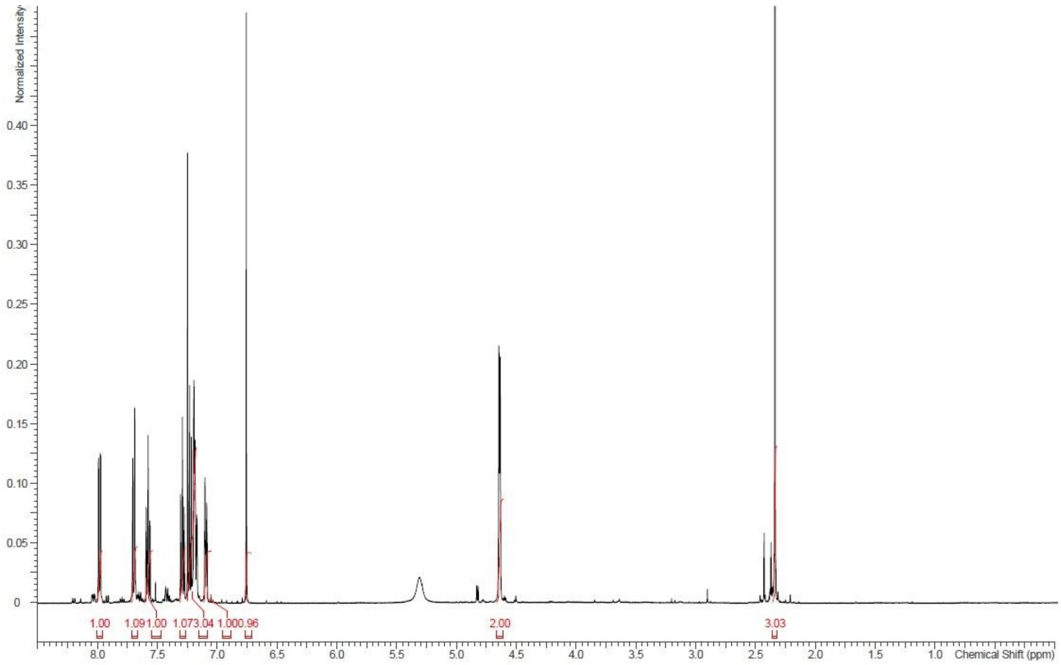

2-Chloro-*N*-(3-chlorobenzyl)quinolin-4-amine (8c)

UPLC-MS

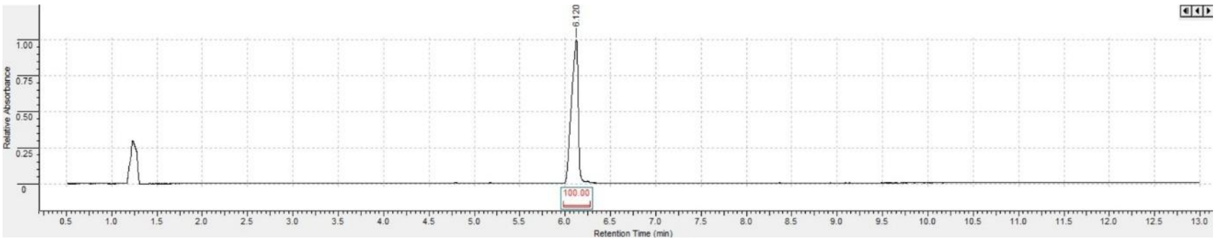

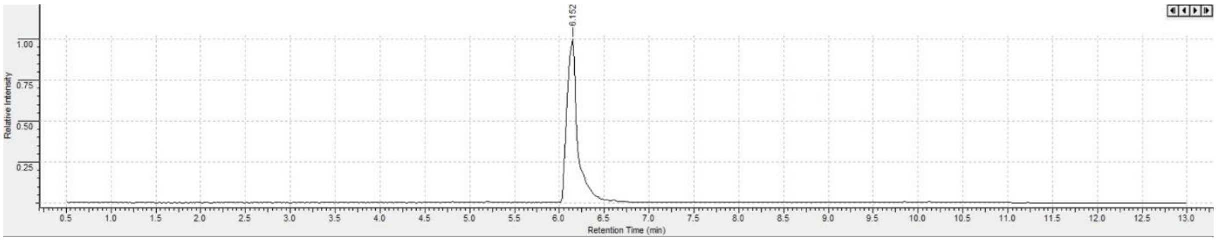

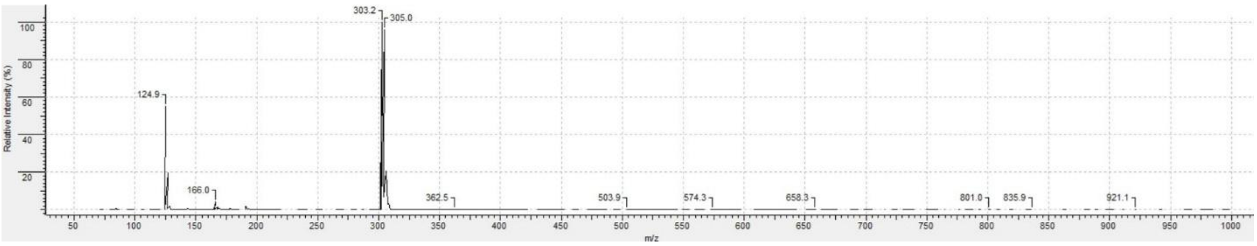

^{1}H NMR

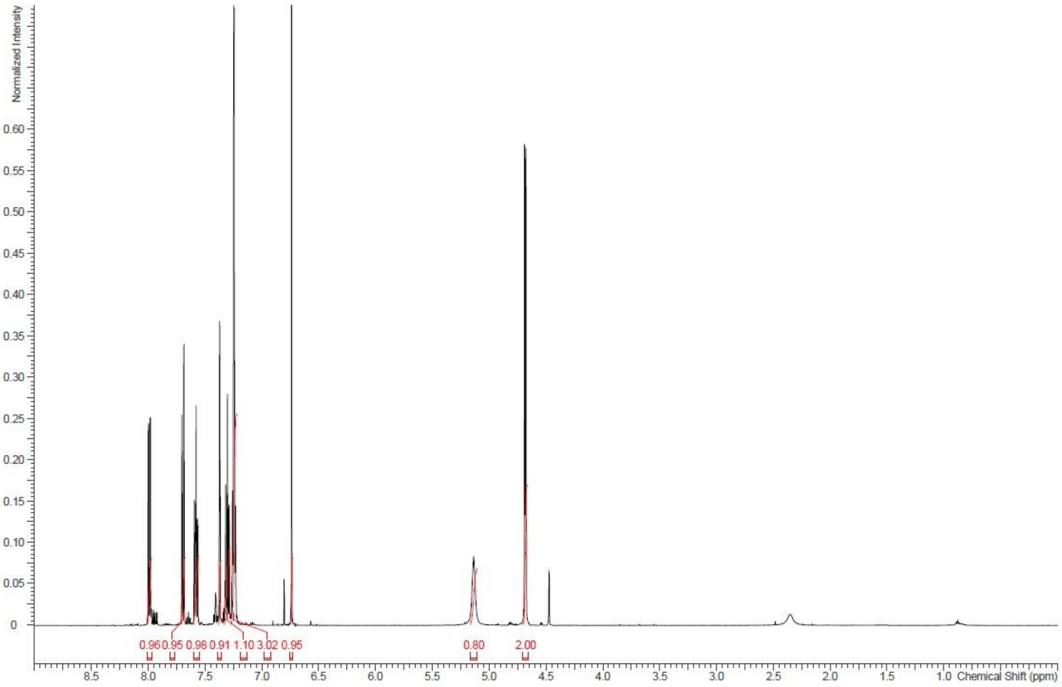

2-Chloro-*N*-(3-chlorobenzyl)-7-methoxyquinolin-4-amine (8e)

UPLC-MS

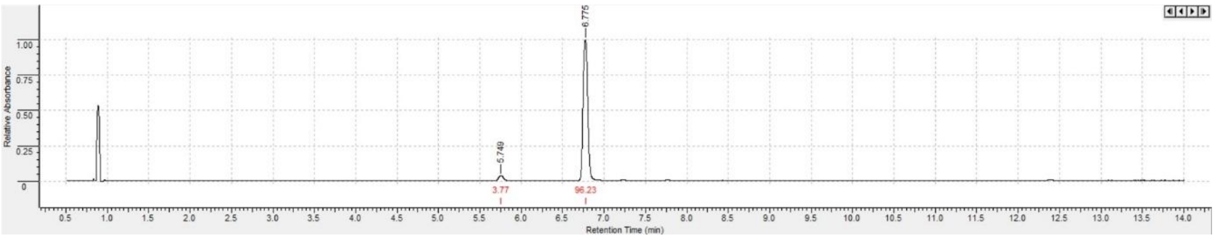

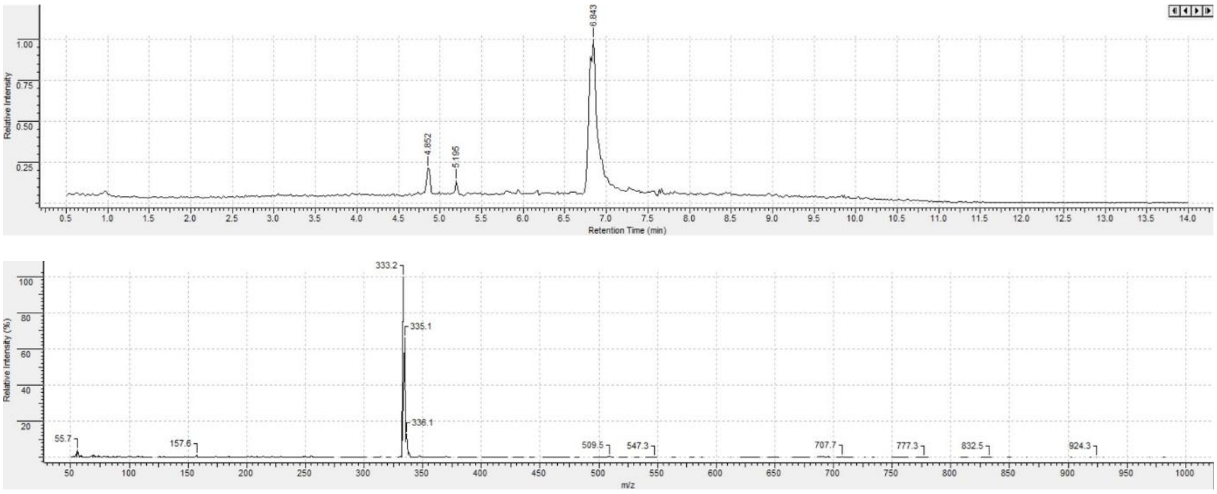

^{1}H NMR

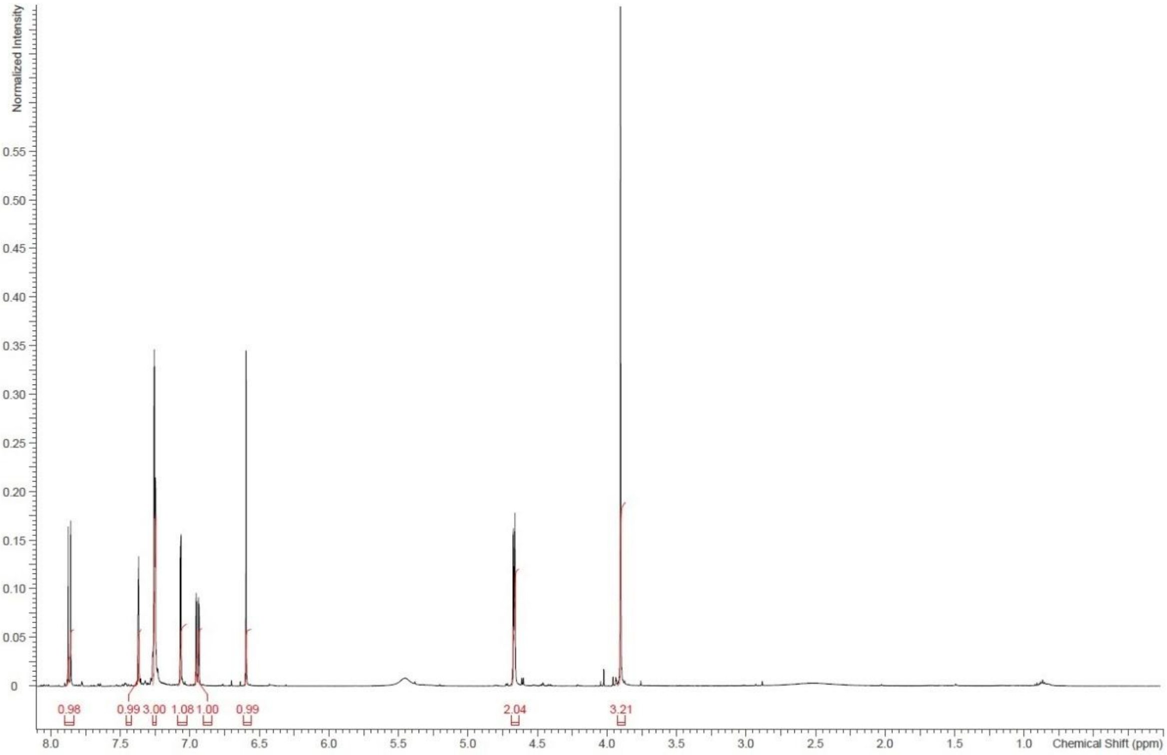

UPLC-MS and ^{1}H NMR and ^{13} C NMR spectra of final compounds:9a-9f and 10

***N*-(3-methylbenzyl)-2-(piperazin-1-yl)quinolin-4-amine hydrochloride (9a)**

UPLC-MS

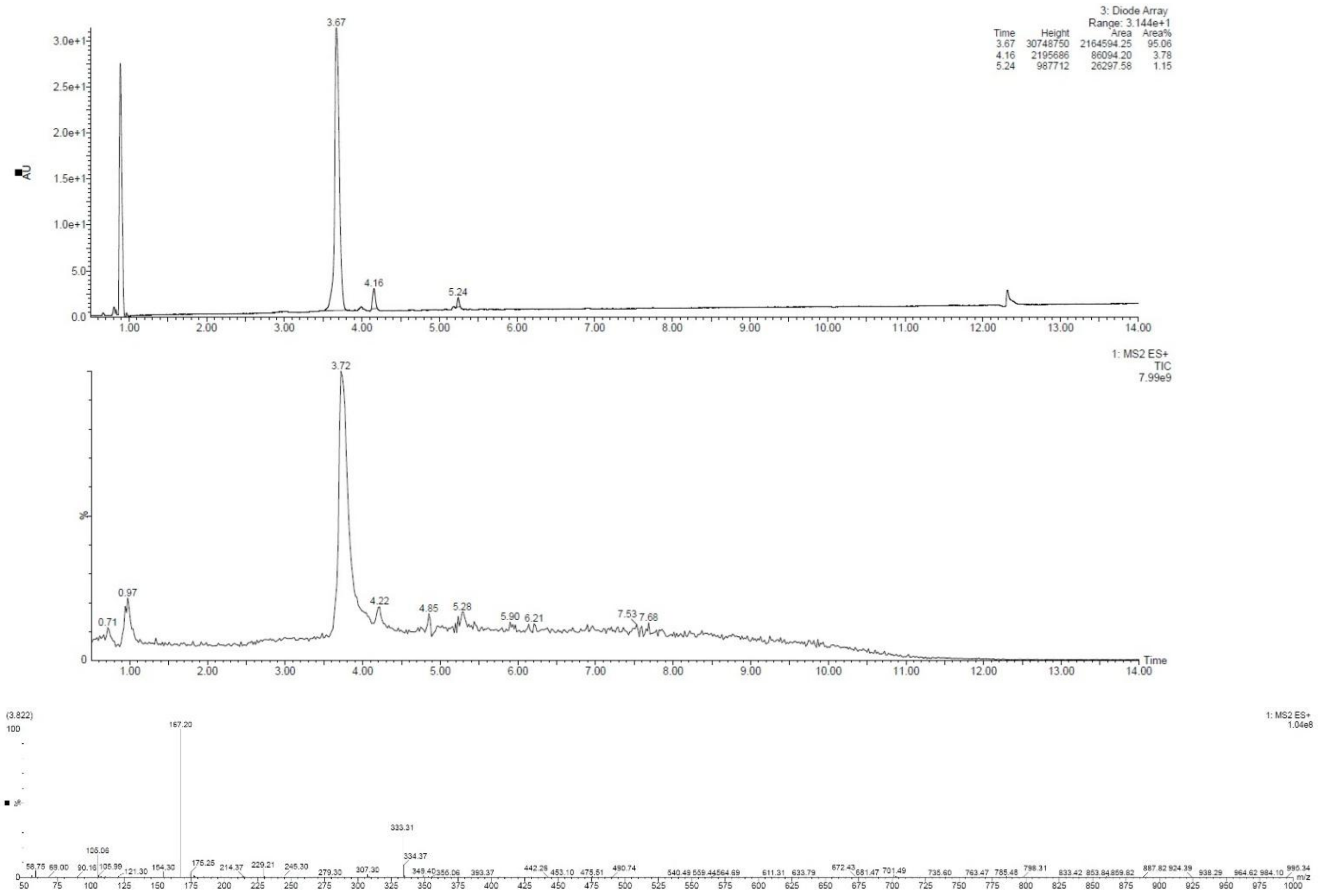

^{1}H NMR

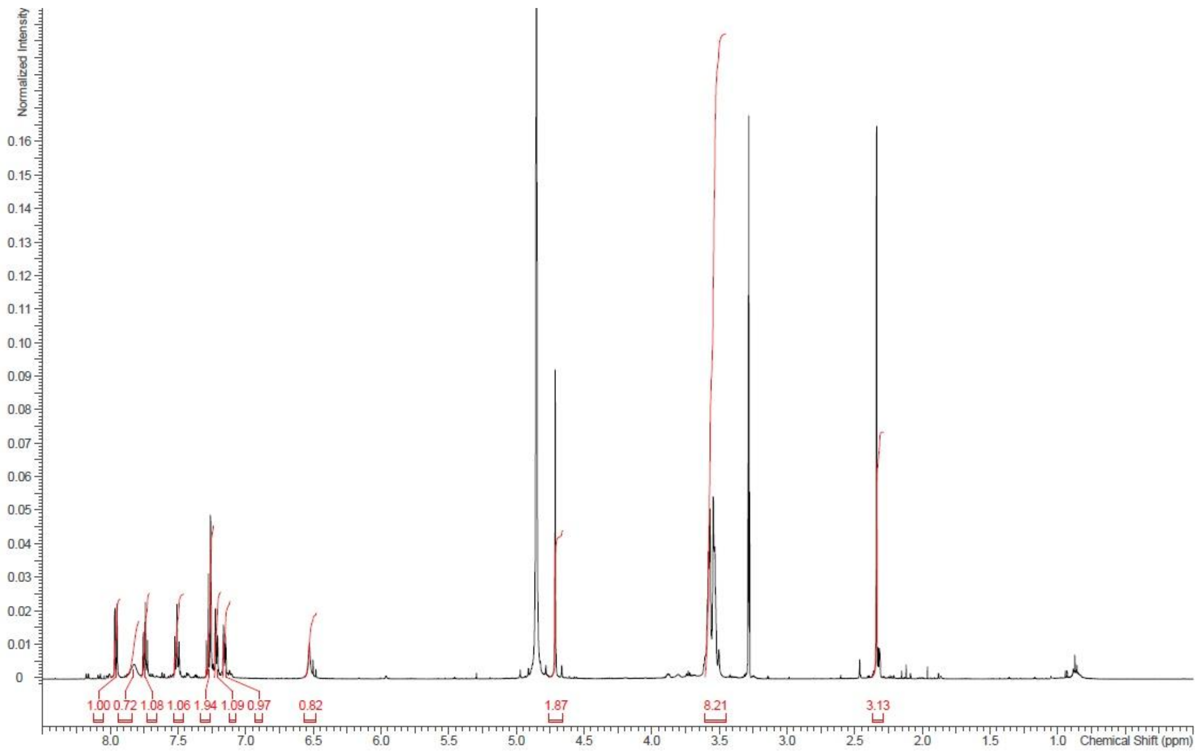
Normalized Intensity
1.00 0.72 1.08 1.06 1.94 1.09 0.97 0.82 1.87 8.21 3.13
Chemical Shift (ppm)

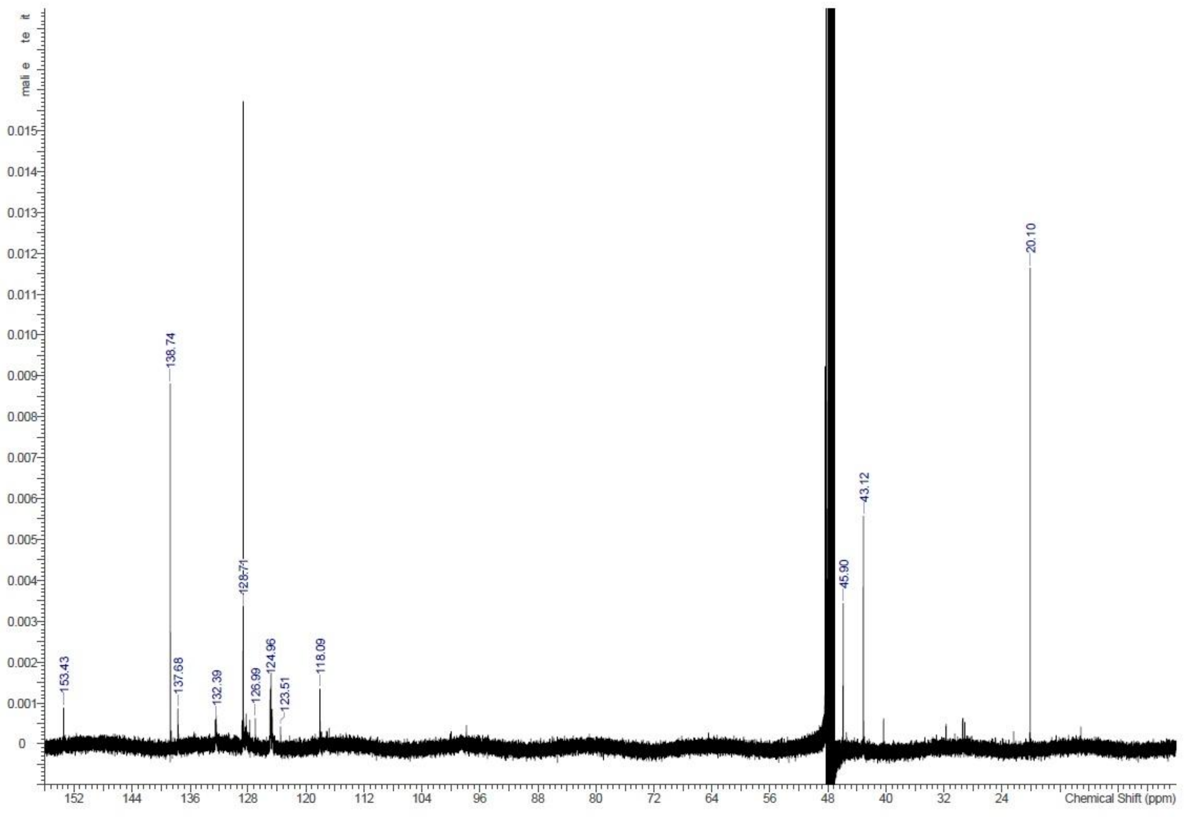
153.43
138.74
137.68
132.39
128.71
126.99
124.96
123.51
118.09
45.90
43.12
20.10
Chemical Shift (ppm)

N-(2-chlorobenzyl)-2-(piperazin-1-yl)quinolin-4-amine hydrochloride (9b)

UPLC-MS

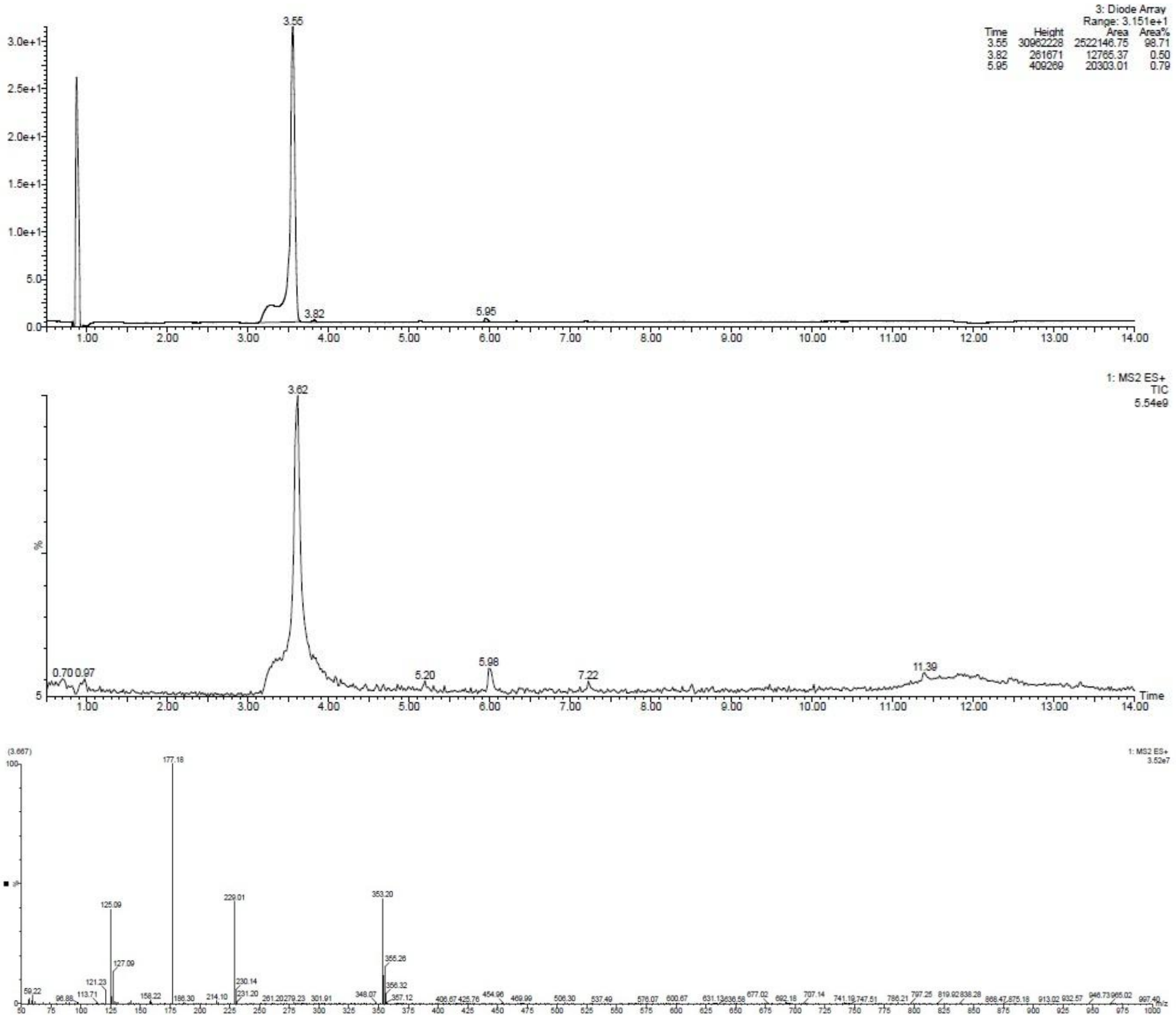

^{1}H NMR

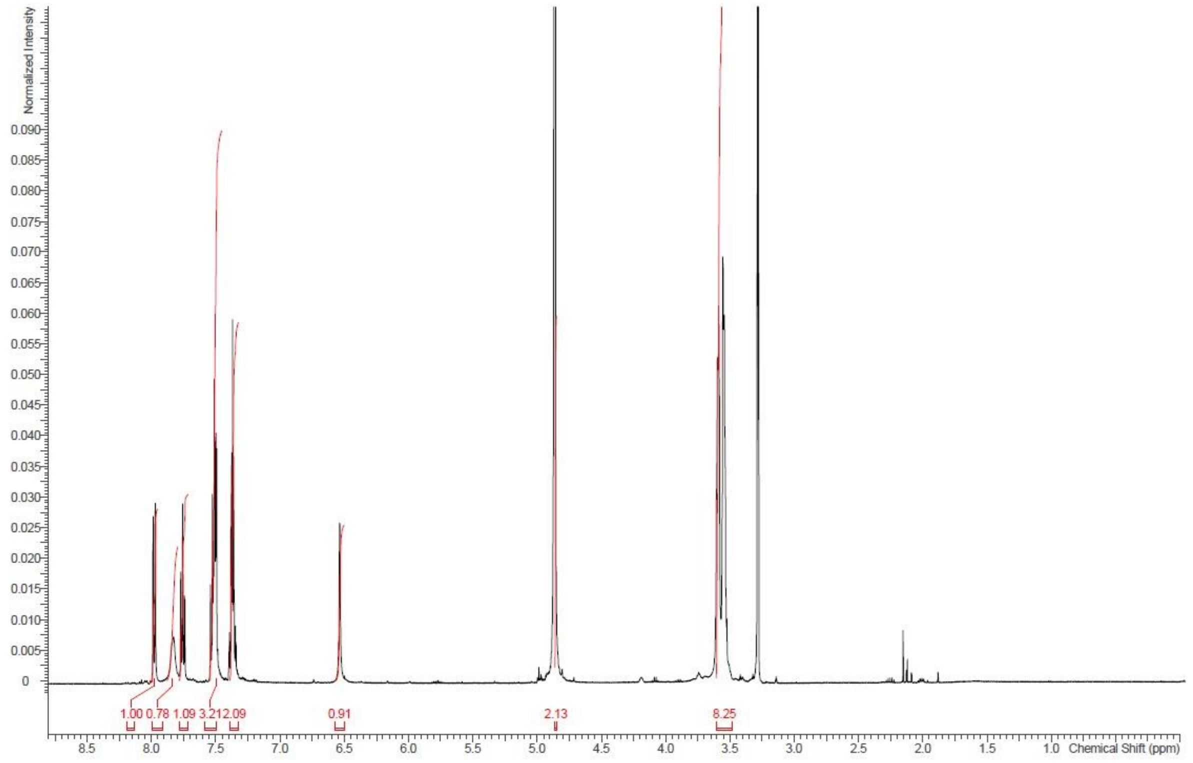

^{13}C NMR

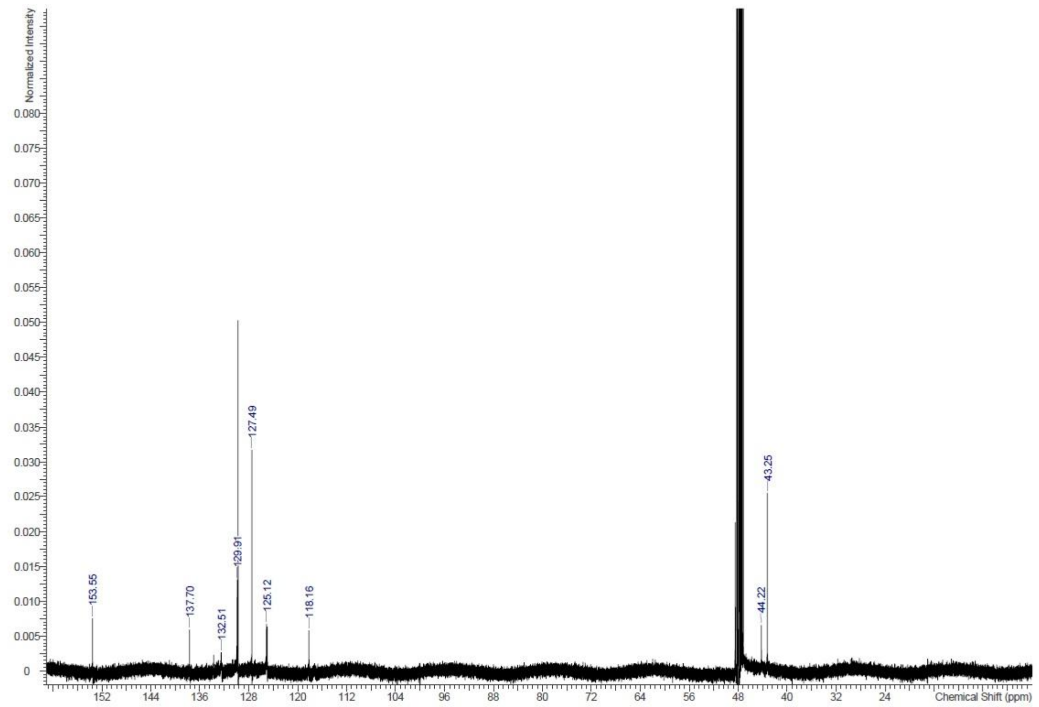

N-(3-chlorobenzyl)-2-(piperazin-1-yl)quinolin-4-amine hydrochloride (9c)

UPLC-MS

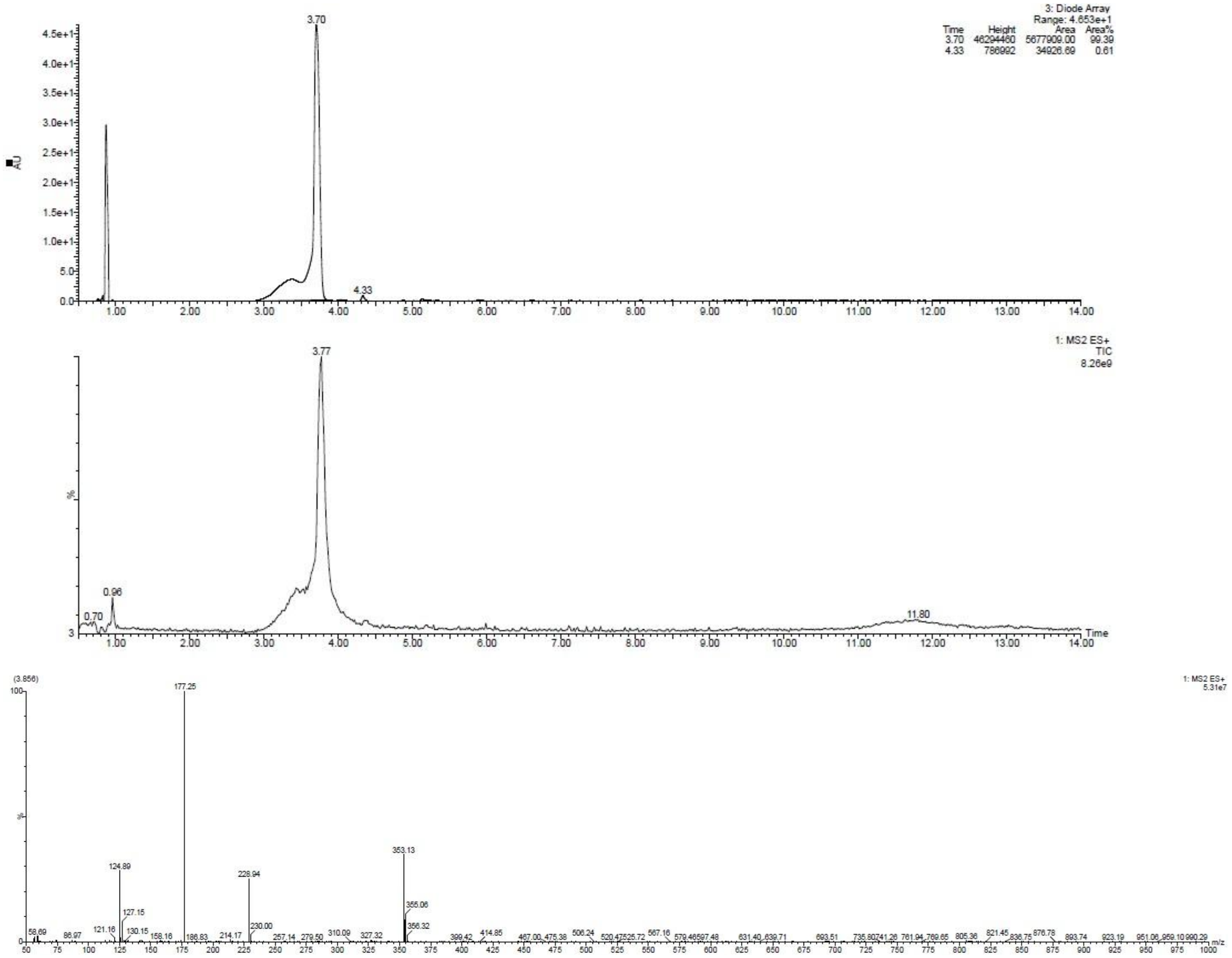

^{1}H NMR

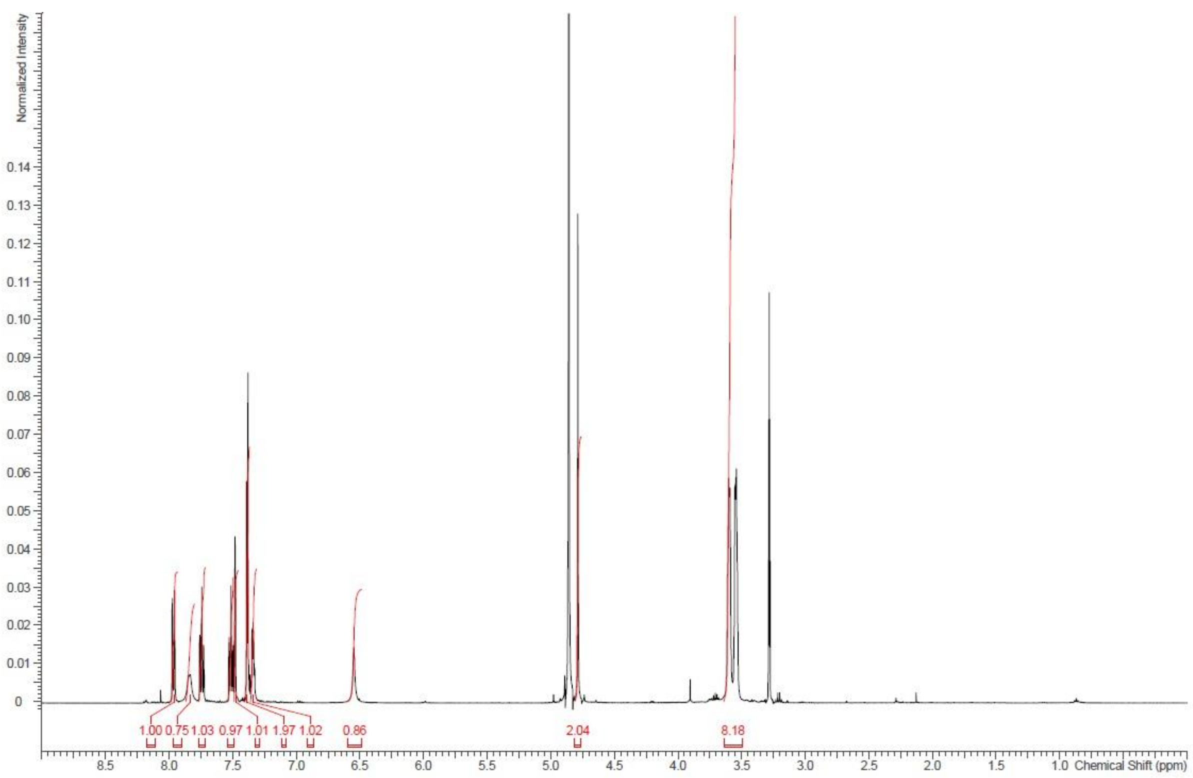

^{13}C NMR

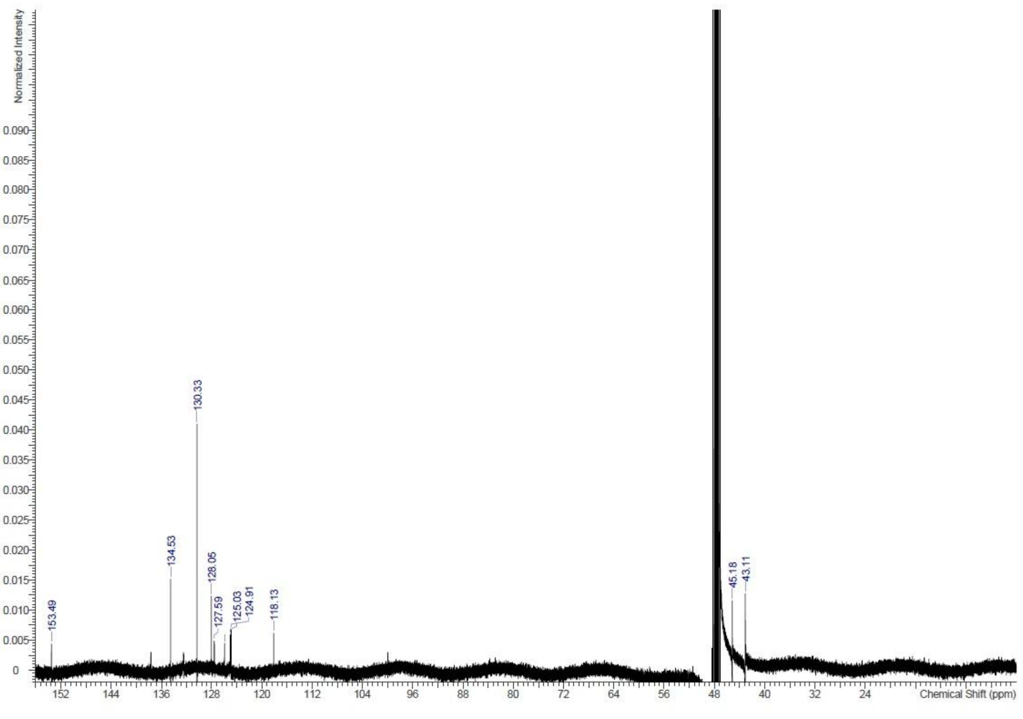

N-(4-chlorobenzyl)-2-(piperazin-1-yl)quinolin-4-amine hydrochloride (9d)

UPLC-MS

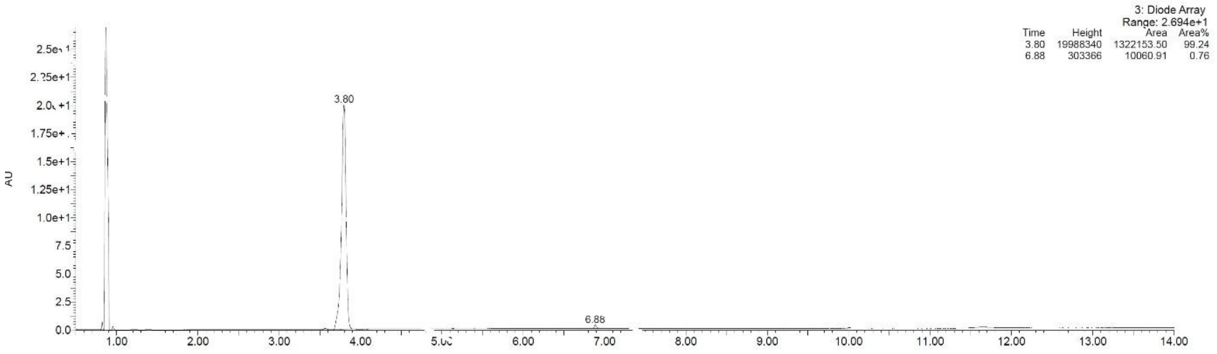

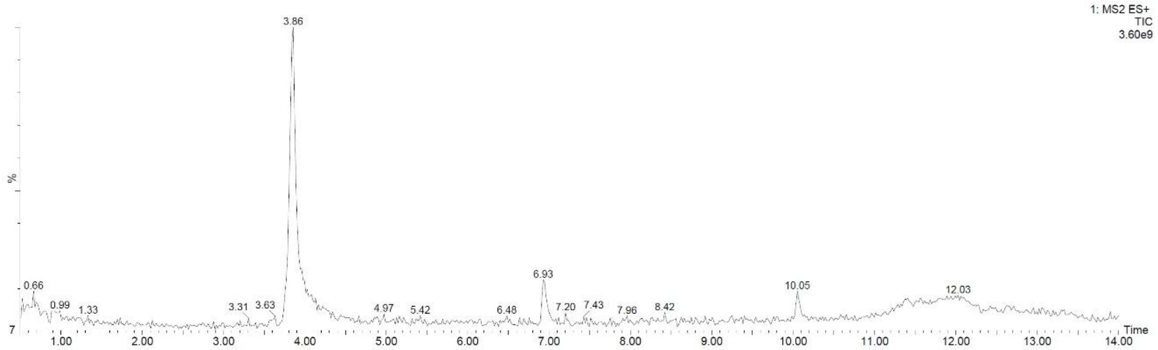

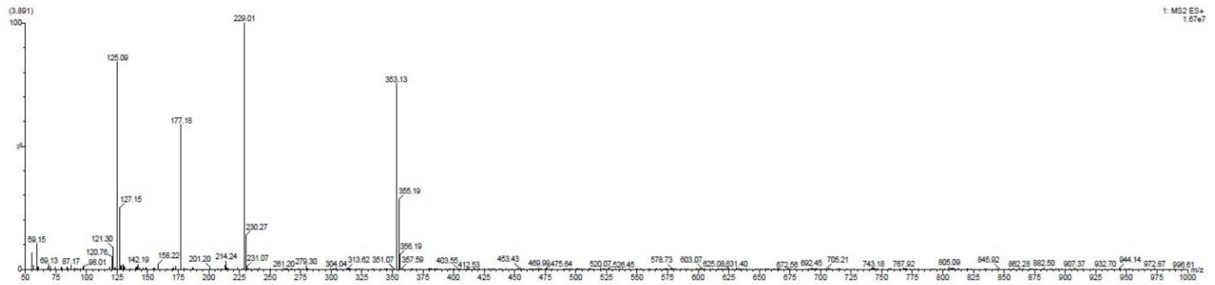

^{1}H NMR

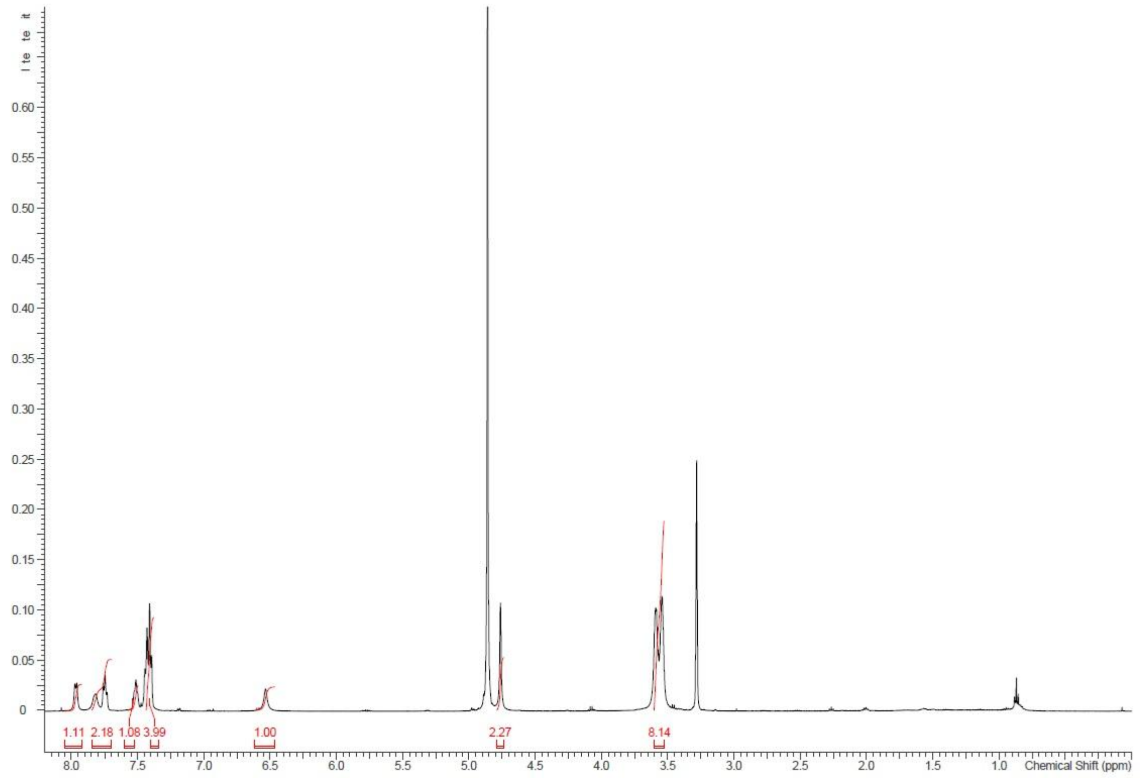

^{13}C NMR

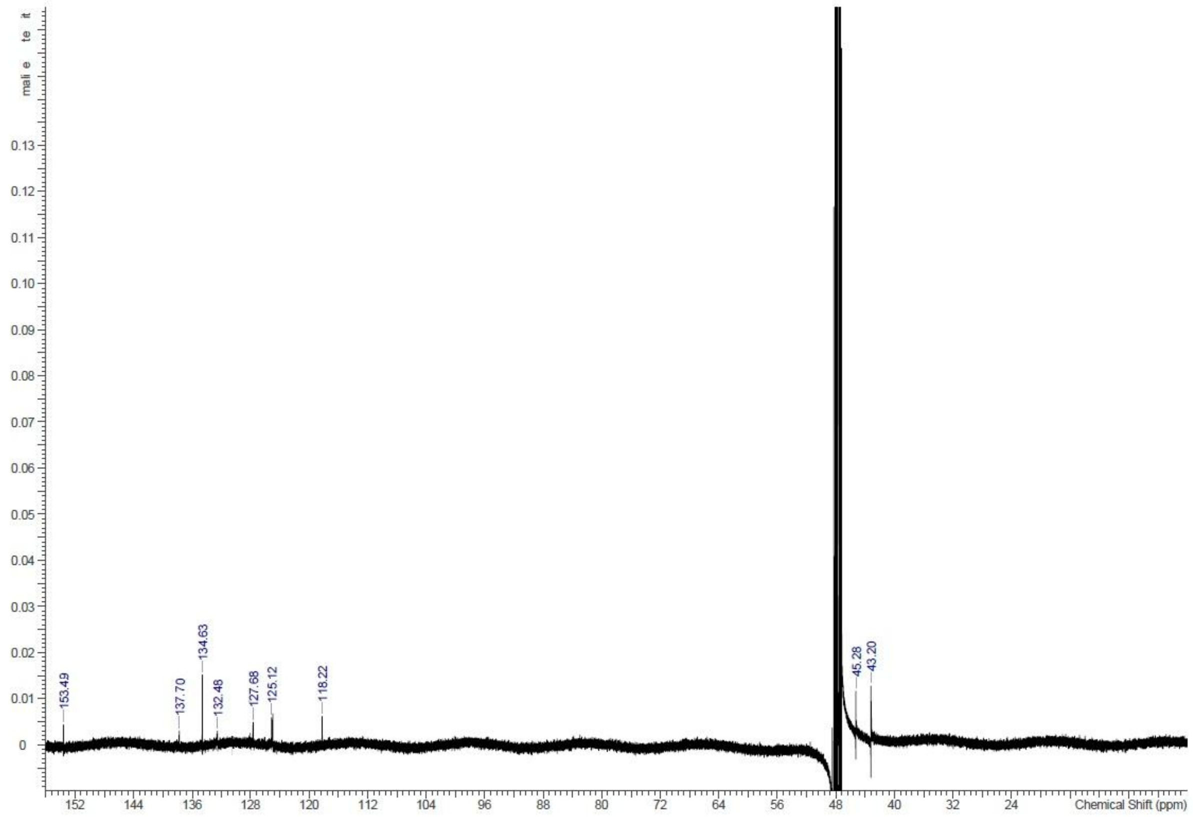

N-(3-chlorobenzyl)-7-methoxy-2-(piperazin-1-yl)quinolin-4-amine hydrochloride (9e)

UPLC-MS

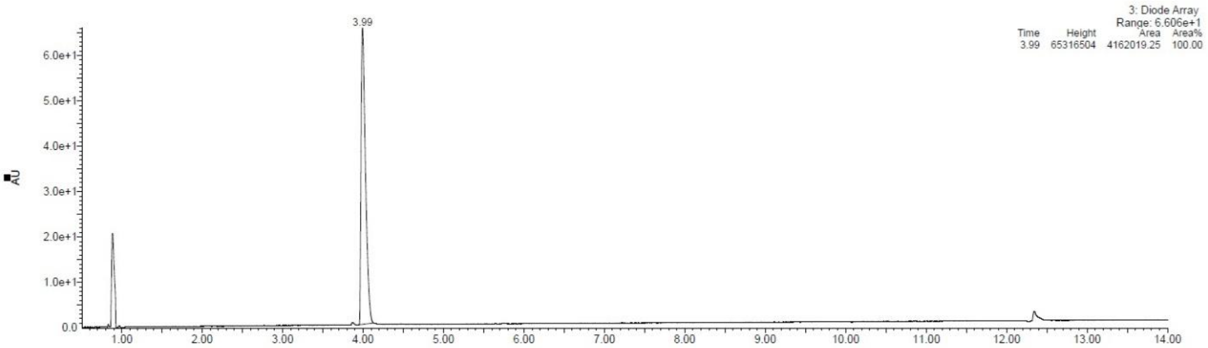

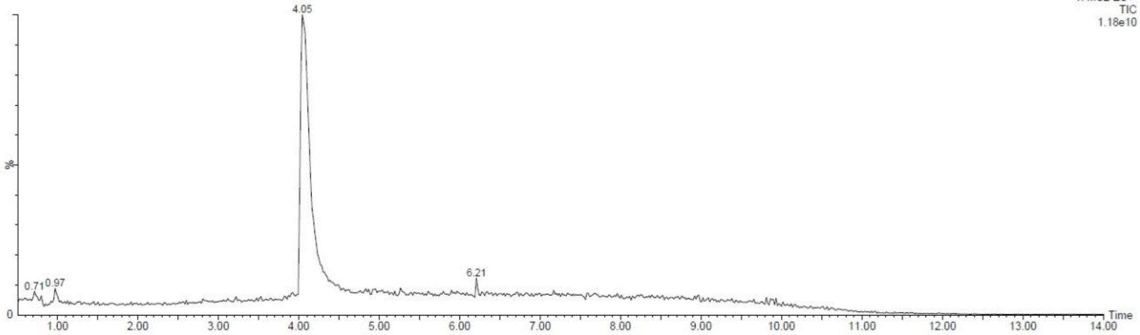

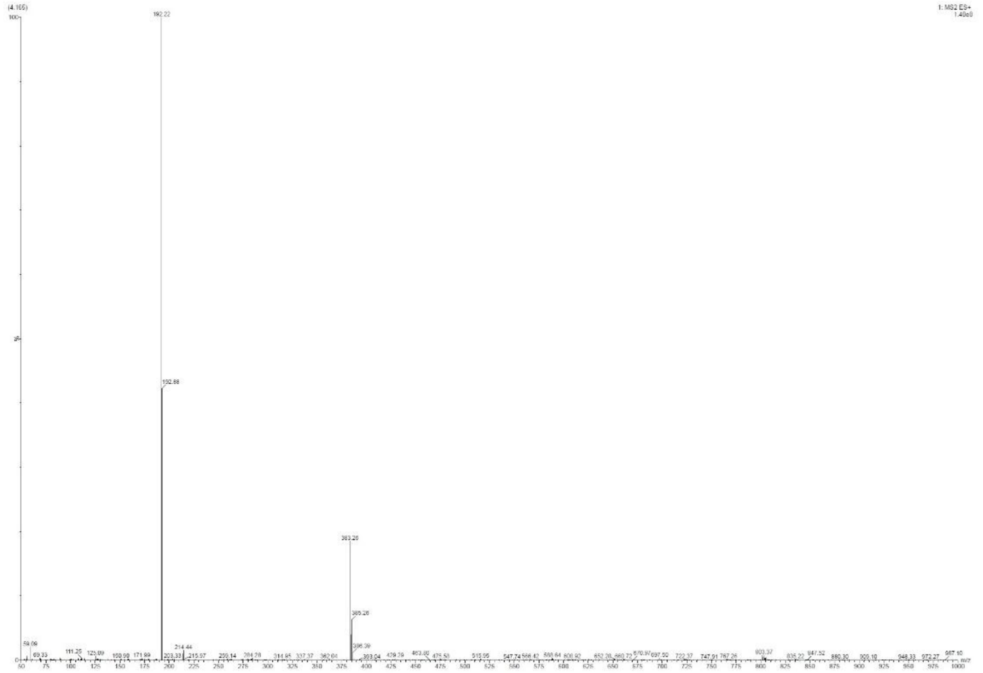

^{1}H NMR

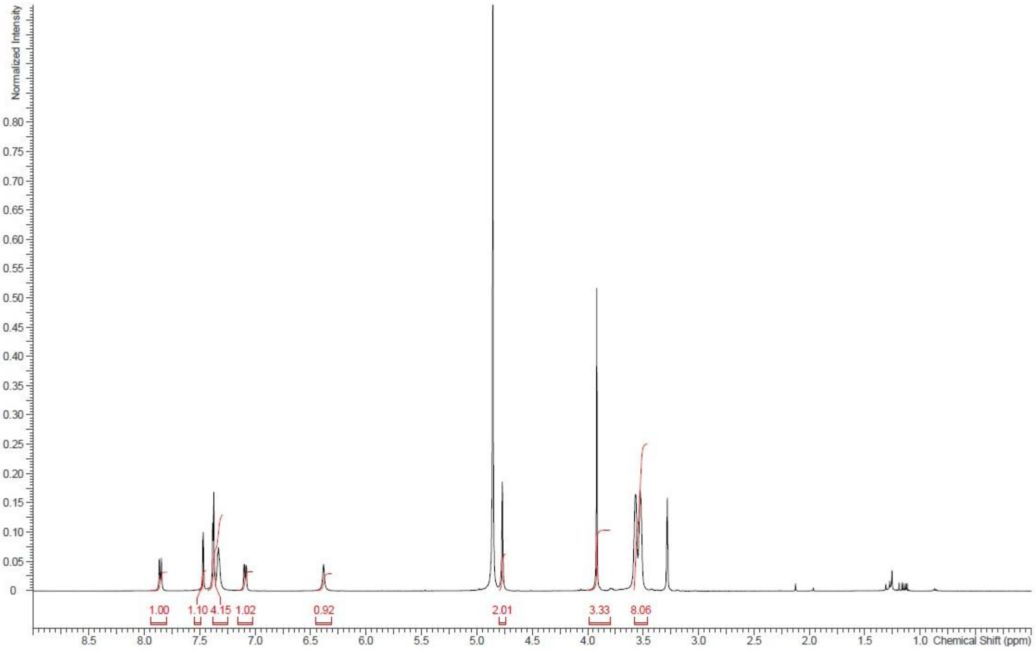

^{13}C NMR

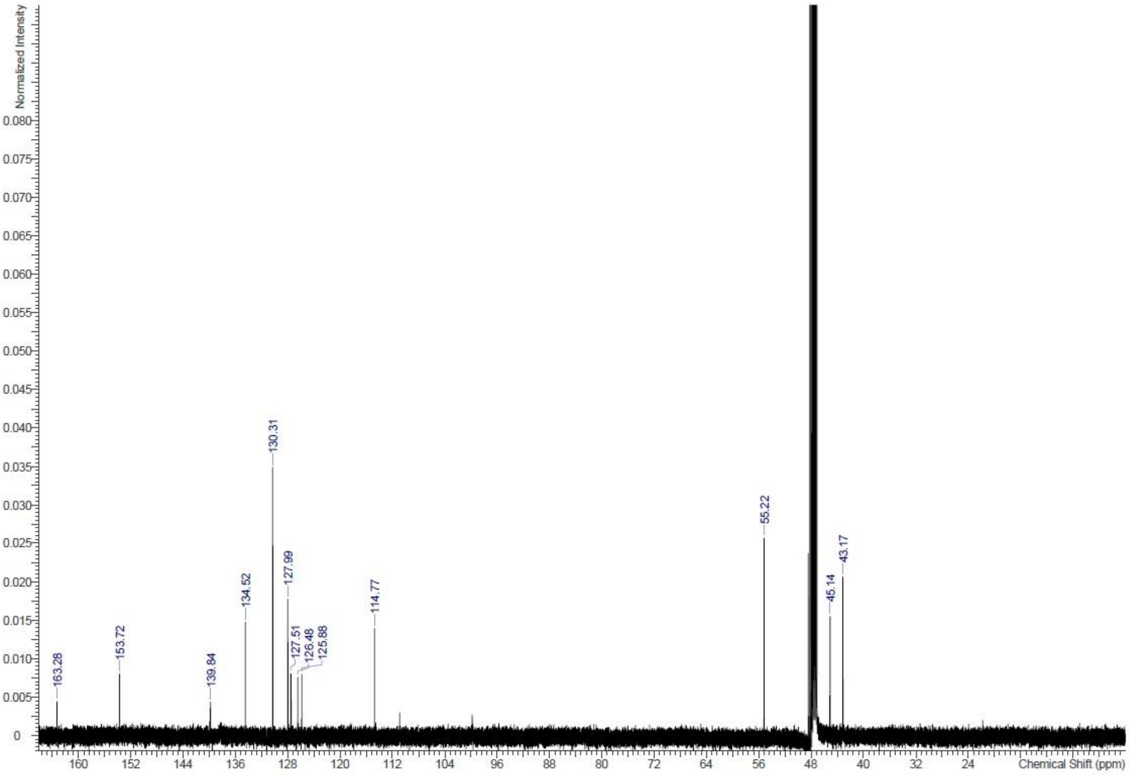

N-(3-chlorobenzyl)-8-methoxy-2-(piperazin-1-yl)quinolin-4-amine hydrochloride (9f)

UPLC-MS

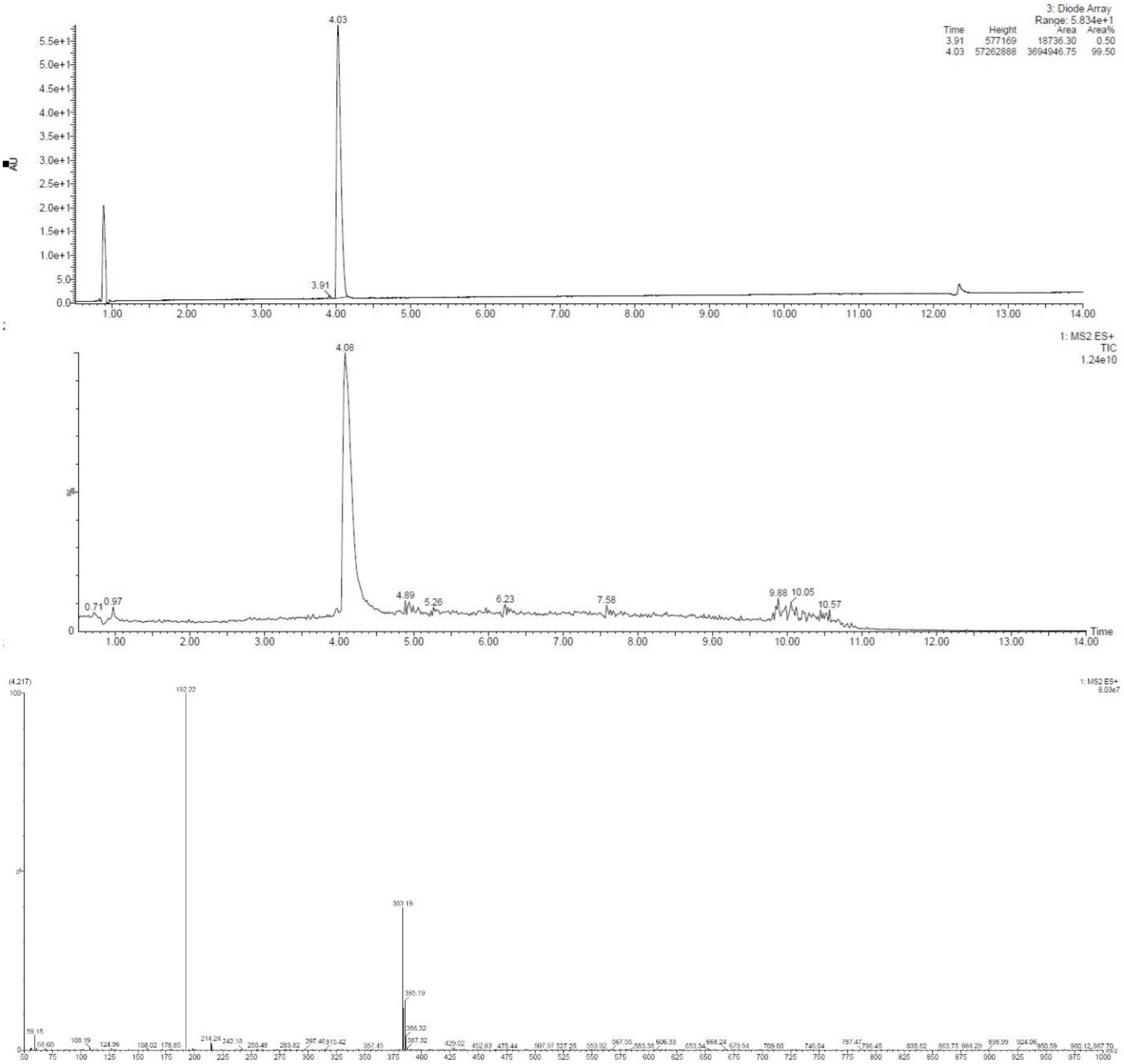

^{1}H NMR

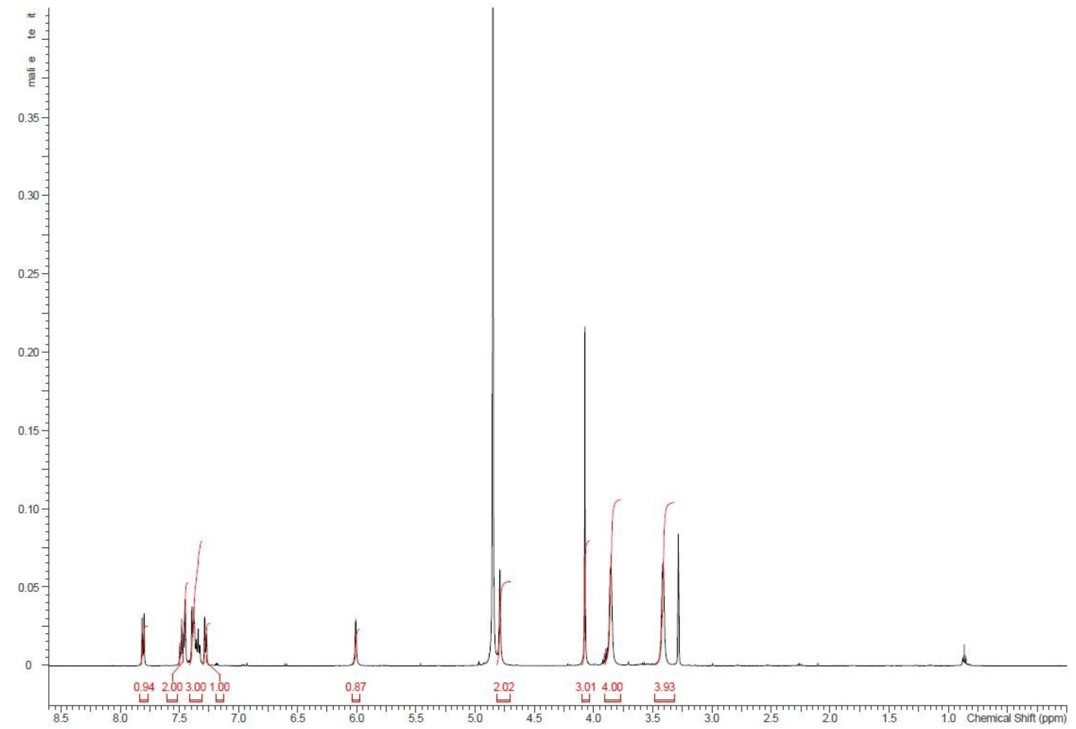

^{13}C NMR

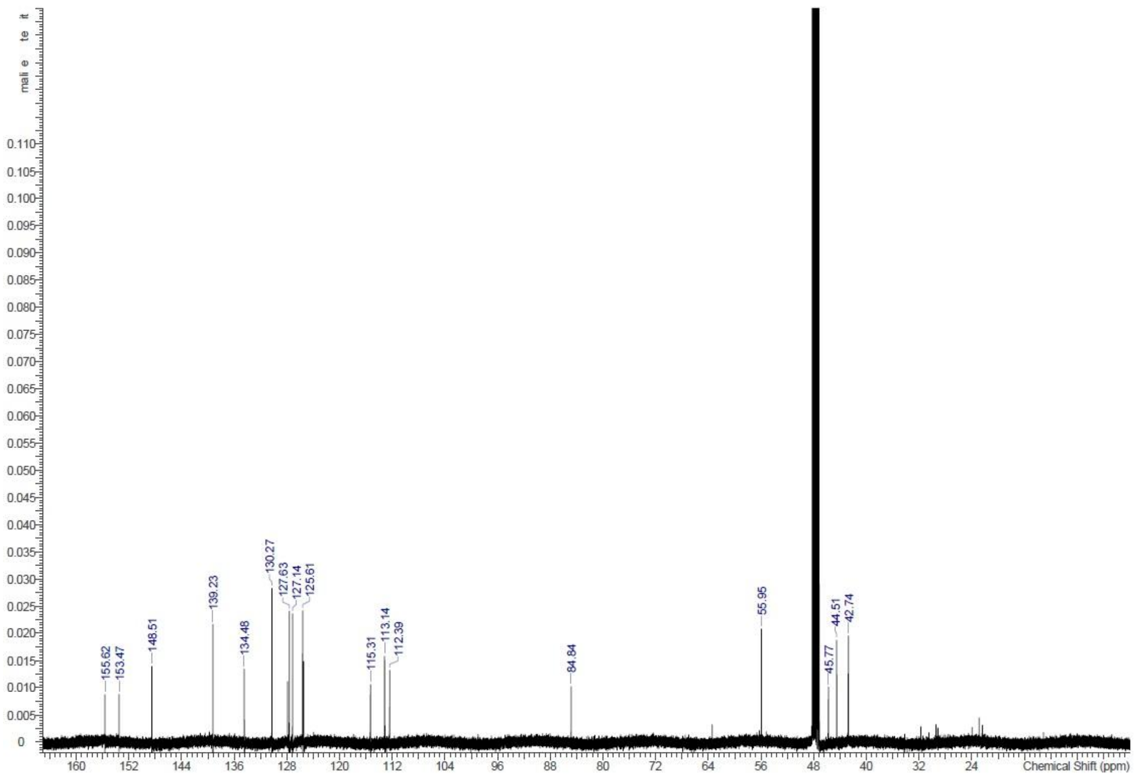

N-(3-chlorobenzyl)-2-morpholinoquinolin-4-amine (10)

UPLC-MS

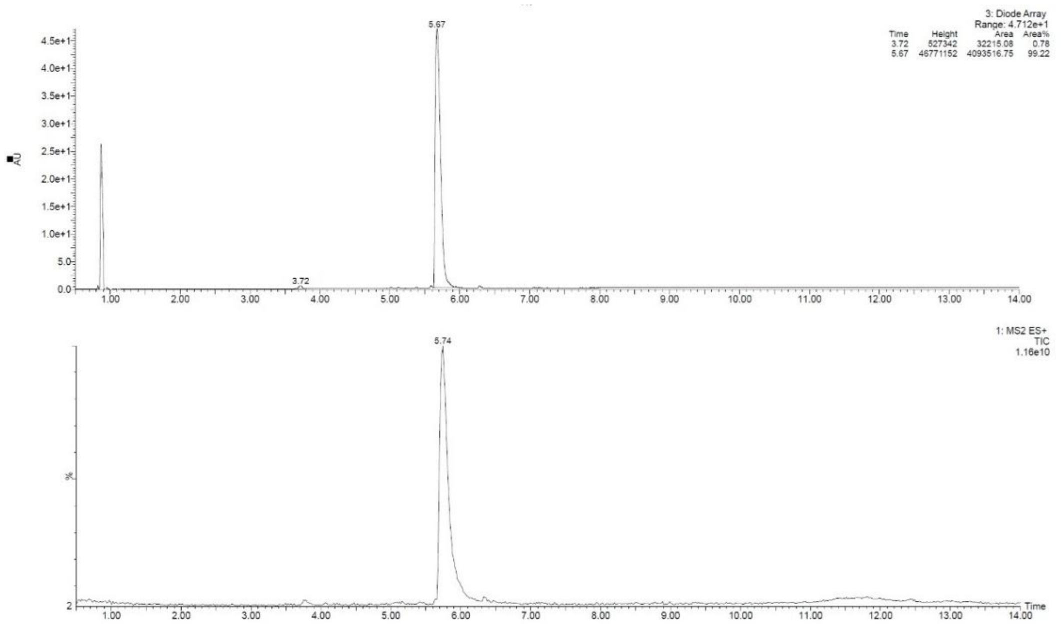

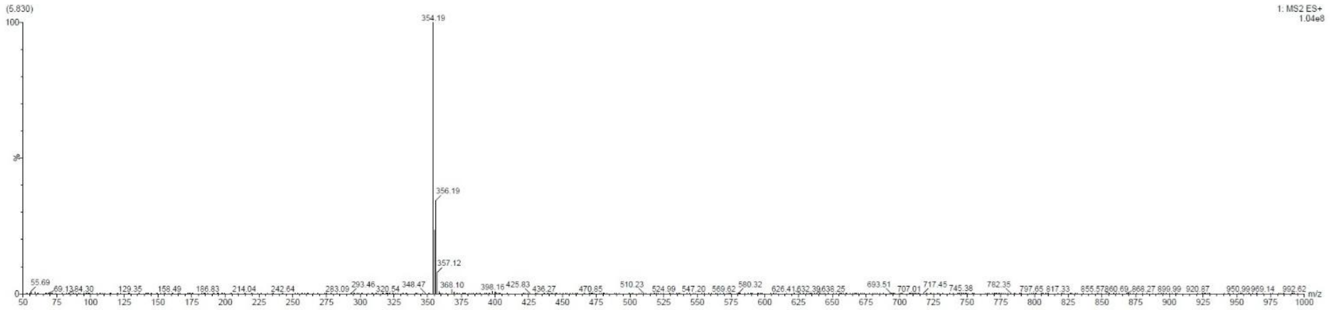

^{1}H NMR

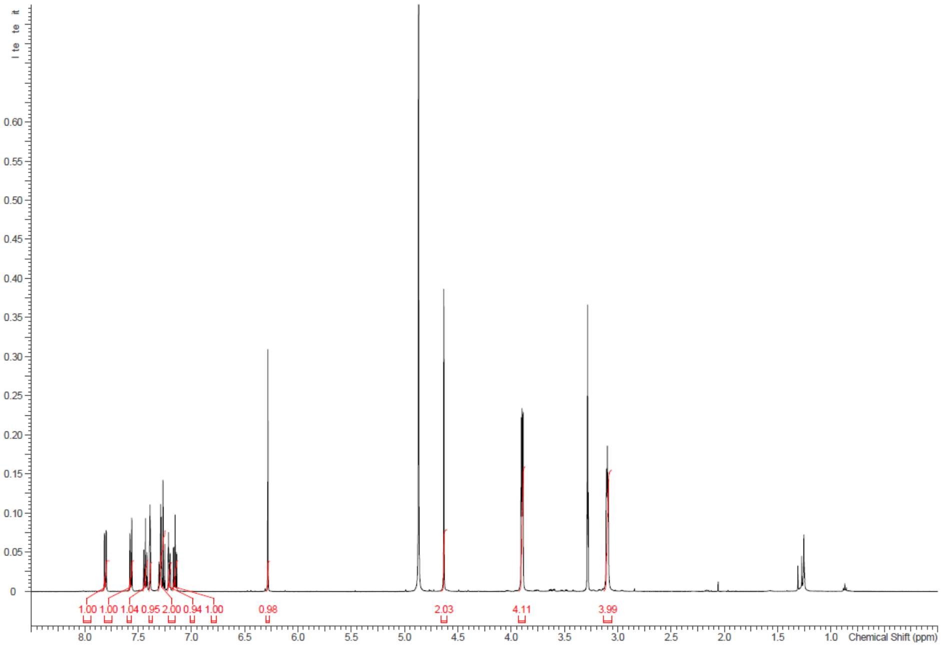

^{13}C NMR

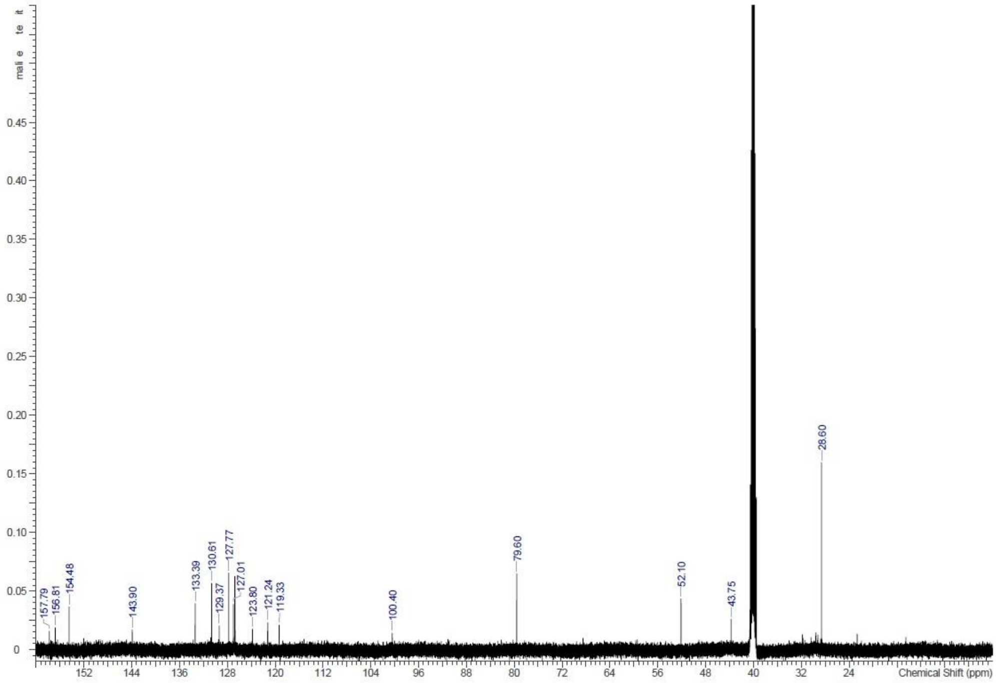

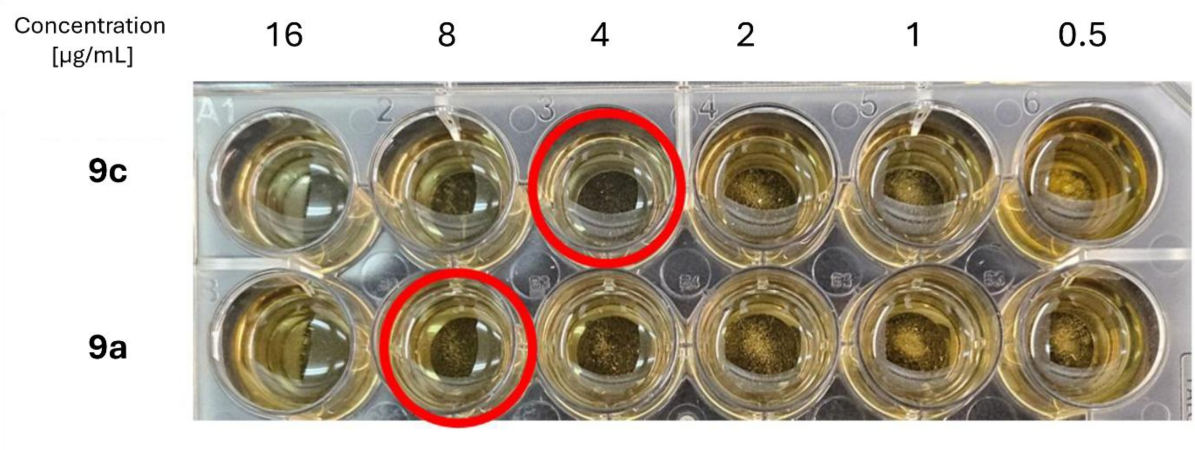

Figure 1-SI. The evaluation of MIC of compounds **9c** and **9a** against reference, metronidazole resistant *H. pylori* ATCC 43504 by broth microdilution method; *Red rings – for MIC values, no visible growth of the bacteria observed.

Impact of 9c on fibroblast viability

For the analysis of the impact of **9c** on fibroblast viability, we have selected concentrations within the estimated MIC range (1.5, 2, 2.5, 3, 4 µg/ml). During the first 24 hours, the addition of **9c** at doses of 1.5 and 2 µg/ml did not induce any significant change in either the total fibroblast counts, or the viable fibroblast count compared to the control (Figure 2-SI.A). The percentage of viable fibroblasts for these doses, as well as for the control, was approximately 90% (Figure 2-SI.B).

At a dose of 2.5 µg/ml, both the total and viable fibroblast counts began to decrease slightly but significantly, as on overall fibroblast viability (Figure 2-SI.A); however, the percentage of viable fibroblasts relative to the total fibroblast count did not differ significantly from the control (Figure 2-SI.B).

For the 3 µg/ml dose, although both the total fibroblast count and the viable fibroblast count were slightly lower compared to the control, these differences were not statistically significant, suggesting that fibroblast proliferation was not markedly affected (Figure 2-SI.A). Nevertheless, the significantly lower percentage of viable fibroblasts relative to the total cell count (approximately 76.5%) points to a negative impact on cell viability (Figure 2-SI.B).

A statistically significant decrease in both total and viable fibroblast counts was observed at 4 µg/ml (Figure 2-SI.A). At this concentration, the decrease in the total fibroblast count, along with the reduction in the number of viable fibroblasts and a lower ratio of viable to dead fibroblasts (59.7%), suggests that the treatment exerts a cytotoxic effect. Specifically, these

results indicate that at 4 µg/ml, **9c** is likely reducing fibroblast proliferation while simultaneously increasing cell death, thereby compromising overall cell viability.

During the following 24 hours, the addition of **9c** at doses of 1.5 and 2 µg/ml again did not induce any significant change in either the total or viable fibroblast counts compared to the control (Figure 2-SI.C). The percentage of viable fibroblasts remained approximately 90% for these doses and for the control (Figure 2-SI.D).

Beginning at 2.5 µg/ml, both the total and viable fibroblast counts began to slightly decrease, indicating a negative influence on overall fibroblast viability; however, the statistically significant decrease in both counts was observed only at the 4 µg/ml dose (Figure 2-SI.C). For this concentration, the percentage of viable fibroblasts dropped to 62.5% (a decrease of approximately 30%), and the total fibroblast count was the lowest measured (Figure 2-SI.D). Although the decrease in proliferation, as assessed by the total fibroblast count and the percentage of viable fibroblasts, was statistically insignificant across doses, there was a tendency for a reduction starting at 2.5 µg/ml. Thus, doses up to 2 µg/ml do not seem to affect fibroblast viability or proliferation. For doses of 2.5 and 3 µg/ml, the slight decrease in both the total number of fibroblasts and the number of viable fibroblasts—while maintaining the ratio of viable fibroblasts to the total fibroblast count—suggests a modest, insignificant reduction in fibroblast proliferation. In contrast, at 4 µg/ml, the total fibroblast count, the viable fibroblast count, and the ratio of viable to dead fibroblasts significantly decreased (Figure 2-SI.D).

The reduction in total fibroblast count, accompanied by a lower ratio of viable to dead fibroblasts (61%), further supports the conclusion that at this concentration, **9c** exerts a cytotoxic effect by reducing proliferation and increasing cell death, ultimately compromising overall fibroblast viability.

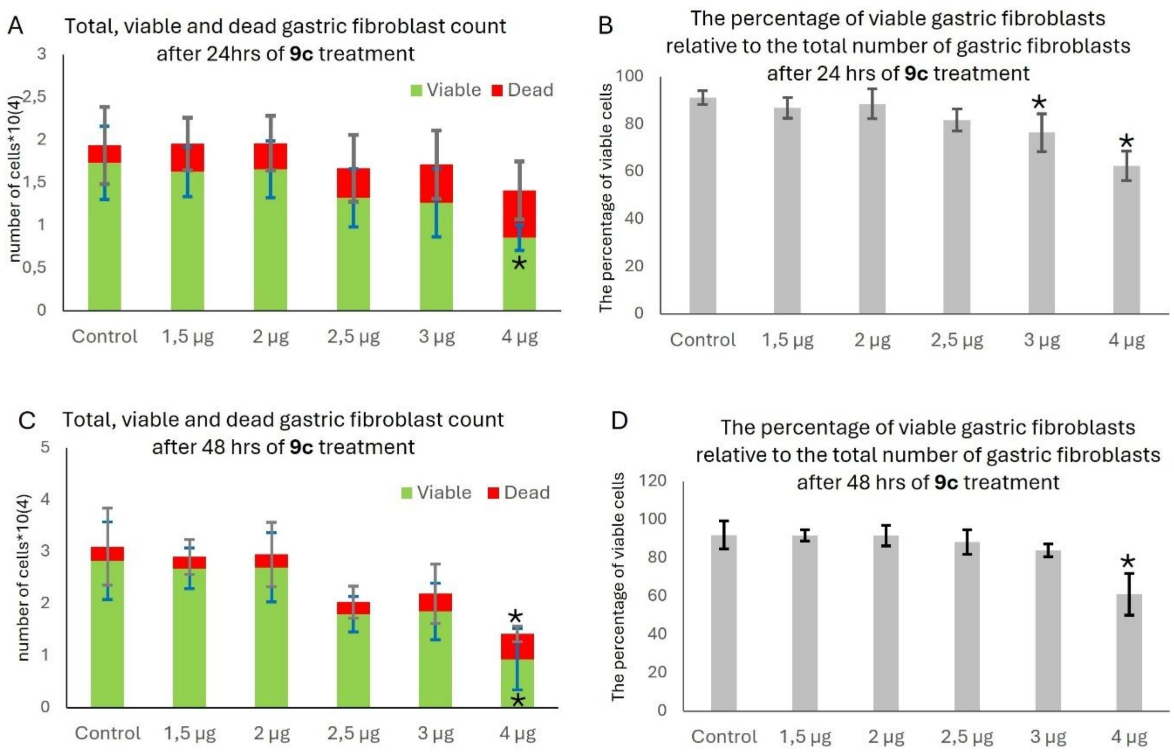

Figure 2-SI. Impact of **9c** on fibroblast viability. (A) The influence of **9c** on the proliferation and viability of normal gastric fibroblasts after 24hrs of incubation. (B) The total dead and viable fibroblast count, the viable fibroblast count, the percentage of viable fibroblast count to the total fibroblast count. (C) The influence of **9c** on the proliferation and viability of normal gastric fibroblasts after 48 hrs of incubation. (D) The total dead and viable fibroblast count, the viable fibroblast count, the percentage of viable fibroblast count to the total fibroblast count. Results are mean ± SEM of six to seven independent experimental repeats. Asterisk (*) indicates a significant change ($P<0.05$) as compared to the control value.

Table 1-SI. Antimicrobial susceptibility profile of tested *H. pylori* strains.

***H. pylori* Strains**	**Characterization**
ATCC 43504	Reference, metronidazole resistant strain, *cagA*+, *vacA* s1m1
ATCC 700684	Reference, clarithromycin resistant strain, *cagA*+, *vacA* s1m2
J99	Reference, susceptible strain, *cagA*+, *vacA* s1m1
3CML	Clinical, multi drug resistant strain (resistant to clarithromycin, metronidazole, levofloxacin), strong biofilm producer, *cagA*+, *vacA* s1m2
1CML (strain used only to anti-biofilm evaluation)	Clinical, multi drug resistant strain (resistant to clarithromycin, metronidazole, levofloxacin), good biofilm producer, *cagA*+, *vacA* s1m2
